# Associations of anthropometric indices with all-cause and cause-specific mortality among US adults with a self-reported history of cancer: a population-based cohort study

**DOI:** 10.1186/s12902-026-02652-w

**Published:** 2026-10-15

**Authors:** Yaxin Li, Peinan Liu, Haoyu Zhu, Danni Zhao, Chuchen Zhao, Xiaobin Ma, Meng Wang, Shuai Lin, Guochao Mao, Huafeng Kang

**Affiliations:** The Comprehensive Breast Care Center, The Second Affiliated Hospital of Xi’an Jiaotong University, Xi’an, 710004 China

**Keywords:** A body shape index, Body mass index, Body roundness index, Cancer history, Mortality, NHANES

## Abstract

**Background:**

Obesity is a well-established risk factor for cancer incidence and mortality. Body mass index (BMI) does not adequately characterize body shape or fat distribution. Whether a body shape index (ABSI) and body roundness index (BRI) are associated with mortality among adults with a history of cancer remains uncertain.

**Methods:**

We analyzed 3,230 NHANES participants (2003–2018) using survey-weighted Cox models adjusted for sociodemographic, behavioral, and cancer-related factors. Nonlinear and sensitivity analyses were performed.

**Results:**

During a survey-weighted median potential follow-up of 7.9 years, 937 all-cause, 315 cancer, and 197 CVD deaths occurred. In the primary model, each 1-SD higher ABSI was associated with all-cause mortality (HR 1.24, 95% CI 1.11–1.38) and CVD mortality (HR 1.55, 95% CI 1.26–1.90), but not cancer mortality (HR 1.12, 95% CI 0.96–1.29). Continuous BRI and BMI were not independently associated with the outcomes. Underweight BMI was associated with higher all-cause mortality (HR 2.44, 95% CI 1.46–4.08). BMI had a nonlinear association with all-cause mortality (P for nonlinearity = 0.002). ABSI findings remained similar across sensitivity analyses.

**Conclusion:**

Higher ABSI was associated with higher all-cause and CVD mortality among U.S. adults with a self-reported history of cancer. Further studies are needed to confirm these associations.

**Clinical trial number:**

Not applicable.

**Supplementary Information:**

The online version contains supplementary material available at https://doi.org/10.1186/s12902-026-02652-w.

## Introduction

Cancer remains a major global health burden. GLOBOCAN 2020 estimated 19.3 million new cancer cases worldwide and projected that this number would increase to 28.4 million annually by 2040 [1]. Obesity prevalence has also increased substantially. In the United States, the age-adjusted prevalence of obesity among adults was 42.4% in 2017–2018 [2]. Higher adiposity has been associated with the incidence of several cancers and with survival outcomes after cancer diagnosis, although these associations vary across cancer types and clinical settings [3–5]. Clarifying how anthropometric characteristics relate to mortality among adults with a history of cancer is therefore epidemiologically important.

Body mass index (BMI) is widely used to classify obesity but cannot distinguish fat mass from fat-free mass or characterize body fat distribution [6]. A Body Shape Index (ABSI) combines waist circumference, BMI, and height and was developed as a body-shape measure complementary to BMI. Higher ABSI has been associated with mortality in general populations [7] and with the incidence of several cancers [8]. The Body Roundness Index (BRI), introduced in 2013, is a geometric index derived from waist circumference and height and was developed to approximate body roundness and correlate with total and visceral adiposity [9]. BRI is linked to various health conditions, such as cardiovascular disease (CVD) [10], diabetes [11], and metabolic syndrome [12]. Evidence regarding BRI in cancer populations remains limited, with available studies focusing on liver cancer incidence and treatment response in early endometrial cancer [13, 14]. Neither ABSI nor BRI directly measures visceral adipose tissue; both should be regarded as anthropometric proxies related to body shape and fat distribution.

Previous studies have evaluated weight-adjusted waist index and mortality among NHANES participants with a history of cancer [15], and ABSI has been examined in relation to prostate cancer-specific mortality [16]. Accordingly, we examined the associations of ABSI, BRI, and BMI with all-cause, cancer-specific, and CVD mortality among U.S. adults with a self-reported history of cancer using data from NHANES 2003–2018. By comparing these three anthropometric indices within a common survey-weighted analytical framework and accounting for cancer type and time since diagnosis, this study aimed to characterize their respective associations with mortality in this population.

## Methods

### Data collection

The National Health and Nutrition Examination Survey (NHANES), conducted by the National Center for Health Statistics (NCHS), is a series of cross-sectional surveys designed to represent the civilian, noninstitutionalized U.S. population through a stratified, multistage probability sampling design [17]. We combined data from eight NHANES cycles conducted from 2003 to 2004 through 2017–2018. Demographic, questionnaire, laboratory, dietary, and mobile examination center (MEC) data were linked by the unique participant identifier. Mortality follow-up was obtained from the public-use 2019 NHANES Linked Mortality File. The NCHS Research Ethics Review Board approved each survey cycle, and all participants provided documented informed consent.

### Study design

This study included adults aged ≥ 20 years with a self-reported history of physician-diagnosed cancer who participated in NHANES 2003–2018. Eligibility for the study cohort was determined by participants’ responses to the survey question: “Has a doctor or other health professional ever informed you that you had cancer or a malignancy of any kind?” Affirmative responses resulted in study inclusion. For those reporting a cancer history, the specific cancer type was further ascertained through the subsequent question: “What kind of cancer was it?” Cancer type was grouped as breast, prostate, non-melanoma/unspecified skin, colorectal, gynecologic, melanoma, hematologic, lung, urinary, or other/unknown. For participants reporting more than one cancer type, the mutually exclusive cancer-group variable used in the multivariable models was based on the first cancer type recorded in the NHANES questionnaire (MCQ230A); this was not necessarily the earliest diagnosed, most recent, or active cancer. The number of reported cancer types was determined from the available responses to MCQ230A–MCQ230D, with two or more reported types classified as multiple cancer diagnoses (Table S10A). Age at first cancer diagnosis was determined separately as the minimum valid diagnosis age across all available cancer diagnoses. Years since first diagnosis equaled age at examination minus age at first diagnosis and was modeled continuously. Among 4,260 adults with a self-reported cancer history, we sequentially excluded 545 participants with incomplete anthropometric data, one without usable mortality follow-up, and 484 with missing covariate data. The primary complete-case analysis therefore included 3,230 participants. The sequential selection process is presented in Figure S1, and variable-specific missingness is reported in Table S10.

### The measurement of obesity indicators

Height, weight, and waist circumference were measured by trained staff in the MEC using standardized NHANES procedures [18]. Waist circumference was measured in a horizontal plane at the level of the uppermost lateral border of the right ilium at the midaxillary line. Body weight was measured using a calibrated digital scale, and standing height was measured using a fixed stadiometer. BMI values were calculated as body weight (kg) divided by height^2^ (m^2^). ABSI was calculated using the equation by Krakauer et al. [7]: $\:ABSI\:=\:\frac{WC}{{BMI}^{2/3}\:\times\:\:{height}^{1/2}}$. BRI was computed by$\:\:364.2\:-\:365.5\:\times\:\:\sqrt{1\:-\:\frac{{(WC/2\pi\:)}^{2}}{{(0.5\:\times\:\:height)}^{2}}}$ [9].

ABSI was standardized using a fixed reference mean of 0.084168552 and standard deviation of 0.004904724. Associations were evaluated per 1-SD increase on this reference scale and across quartiles defined using raw ABSI values in the final analytic sample. BRI was analyzed per 1-unit increase and by quartiles. ABSI and BRI quartile cut points were derived from their raw values in the final analytic sample. BMI was analyzed as a continuous variable per 1 kg/m² increase and categorized as underweight (< 18.5 kg/m²), normal weight (18.5 to < 24.0 kg/m²), overweight (24.0 to < 28.0 kg/m²), or obesity (≥ 28.0 kg/m²).

### Measurements of mortality

Mortality status and follow-up time were obtained from the public-use NHANES Linked Mortality File through December 31, 2019 [19]. Follow-up time was calculated from the date of the MEC examination to the date of death or the end of mortality follow-up using the variable PERMTH_EXM. All-cause mortality was defined as death from any cause. Cancer-specific mortality and CVD mortality were defined based on the public-use leading-cause-of-death classification. In cause-specific Cox models, deaths from causes other than the outcome of interest were censored at the time of death.

### Covariates

Potential confounders were selected on epidemiologic grounds, with the assumed relationships illustrated in the directed acyclic graph in Figure S3. The primary adjustment set included age, sex, race/ethnicity, education level, poverty-income ratio, marital status, Alcohol status, smoking status, cancer group, and years since first cancer diagnosis.

Participants were categorized by smoking history: “never smokers” (fewer than 100 cigarettes in their lifetime), “former smokers” (≥ 100 cigarettes smoked, not current) and “current smokers” (≥ 100 cigarettes smoked, smoking occasionally/daily). Alcohol use was derived from the Alcohol Use Questionnaire and indicated any drinking during the past 12 months; participants reporting no past-year drinking or fewer than 12 lifetime drinks were classified as non-users according to cycle-specific questionnaire logic. Hypertension was defined as physician-diagnosed hypertension, self-reported antihypertensive medication use, or blood pressure ≥ 140/90 mmHg. Diabetes was defined by self-reported diagnosis, glycated hemoglobin A1c ≥ 6.5%, or current use of antihyperglycemic medication. CVD was defined by a self-reported history of any of the following (NHANES questionnaire items): MCQ160B (congestive heart failure), MCQ160C (coronary heart disease), MCQ160D (angina), MCQ160E (heart attack), MCQ160F (stroke). Because hypertension, diabetes, and CVD history may partly lie on pathways linking adiposity to mortality, they were not included in the primary confounder-adjusted model. Instead, they were added in an extended cardiometabolic sensitivity model [20].

### Statistical analysis

Survey-weighted analyses accounted for the complex NHANES sampling design, including strata (SDMVSTRA) and primary sampling units (SDMVPSU). A combined 16-year MEC examination weight (WTMEC16YR) was constructed by dividing the original 2-year MEC examination weight (WTMEC2YR) by eight to account for the eight survey cycles from 2003 to 2004 through 2017–2018 [17]. Baseline continuous variables are survey-weighted means with standard deviations and categorical variables are unweighted counts with survey-weighted percentages. Group comparisons used survey-weighted linear regression or Rao-Scott chi-square tests.

Median potential follow-up was estimated using a survey-weighted reverse Kaplan–Meier curve fitted with survey::svykm. The reverse survival process was specified as Surv(PERMTH_EXM/12, 1 − MORTSTAT), in which deaths were treated as censored observations and administrative censoring of participants who remained alive at the end of mortality follow-up was treated as the event. The survey design incorporated WTMEC16YR, SDMVSTRA, and SDMVPSU. The median and interquartile range were obtained from the estimated potential-follow-up distribution. Survey-weighted Cox models estimated hazard ratios (HRs) and 95% confidence intervals (CIs). Model 1 was unadjusted. Model 2 adjusted for Age, Sex, Race/ethnicity. Model 3 adjusted for Age, Sex, Race/ethnicity, Education level, PIR, Marital status, Alcohol status, Smoking status, cancer group, and years since first cancer diagnosis. Model 4 additionally adjusted for hypertension, diabetes, and CVD history and was treated as an extended cardiometabolic sensitivity model.

Restricted cubic spline models used four survey-weighted knots at the 5th, 35th, 65th, and 95th percentiles, with the survey-weighted median as the reference [21]. Overall and nonlinear P values were reported. The proportional-hazards assumption was assessed using scaled Schoenfeld residual diagnostics by applying survival::cox.zph directly to each survey-weighted survey::svycoxph Model 3 fit (transform = “km”, terms = TRUE); no separate unweighted Cox model was fitted. Diagnostics were examined for the primary exposure, each model term, and the global test. Because cox.zph is not a survey-design-calibrated diagnostic procedure, the resulting P values were interpreted as approximate, exploratory residual diagnostics [22]. Supporting sensitivity models were stratified by covariates identified in the exploratory diagnostics, allowing separate baseline hazard functions across strata. In the all-cause mortality models, years since first cancer diagnosis was categorized as < 5, 5–<10, and ≥ 10 years for stratification, and its continuous adjustment term was omitted. Years since first cancer diagnosis remained continuous in the primary models. To assess potential reverse causation, we performed 12- and 24-month landmark analyses. Participants who died or were censored at or before the corresponding landmark were excluded. Among participants who remained alive and under observation beyond the landmark, follow-up time was reset to zero and accrued from the landmark until death or administrative censoring through December 31, 2019.

For the competing-risk sensitivity analyses, survival::finegray was used to expand the risk sets for cancer-specific and CVD mortality and to generate the Fine–Gray weights (fgwt). The final analysis weight was calculated as WTMEC16YR × fgwt. The expanded data were analyzed using survey::svycoxph within a survey design specified with SDMVSTRA as the sampling strata and SDMVPSU as the primary sampling units. Variance estimates were obtained using the design-based linearization procedure implemented in the survey package. Estimates are reported as subdistribution hazard ratios (sHRs). Because this two-stage approach is not a dedicated Fine–Gray procedure developed specifically for complex survey data, it was interpreted as an approximate survey-weighted competing-risk sensitivity analysis [23]. Exploratory subgroup analyses used formal interaction tests. Post hoc cancer-group analyses used survey-weighted Model 3 models with indicator-by-cancer-group interactions.

Group-specific hazard ratios were estimated using linear contrasts with common covariate effects because separate within-group models were unstable. ABSI scaling was consistent across groups. Global interactions were assessed using design-based Wald tests. Benjamini–Hochberg correction was applied to 36 ABSI subgroup interaction tests and, separately, nine cancer-group interaction tests; these corrections did not provide study-wide multiplicity control. Within-group P values were unadjusted and interpreted descriptively. Sensitivity analyses repeated Model 3 after excluding participants reporting non-melanoma or unspecified skin cancer, while retaining those with melanoma. As a sensitivity analysis, covariate-only multiple imputation by chained equations was performed among 3,714 participants with complete anthropometric measurements and eligible mortality follow-up. Twenty imputed datasets were generated with 10 iterations [24]. Missingness and sequential exclusions are reported in Table S10. Exposures, mortality outcomes, follow-up time, and survey-design variables were not imputed, and years since first cancer diagnosis was recalculated after imputation. Survey-weighted Cox estimates were pooled using Rubin’s rules. Analyses used R version 4.4.1. Tests were two-sided; *P* < 0.05 was considered statistically significant, with multiplicity-adjusted q values emphasized for subgroup interactions.

## Results

### Baseline characteristics of study participants

The final sample included 3,230 participants with a self-reported history of cancer: 635 normal-weight, 49 underweight, 939 overweight, and 1,607 participants with obesity. Weighted mean years since first cancer diagnosis did not differ materially across BMI groups (*P* = 0.181), whereas cancer-group composition differed (*P* = 0.002). BMI groups also differed by sex, poverty-income ratio, smoking, hypertension, and diabetes; age category, education, marital status, alcohol use, and CVD history were not statistically different at the 0.05 level **(**Table 1**)**. Additionally, supplementary tables (Table S1 and Table S2) provide further stratification by ABSI and BRI quartiles, offering more details on body composition and its association with demographic and clinical characteristics.

**Table 1:** Survey-weighted baseline characteristics by BMI category among participants (*N* = 3,230)

Characteristic	Body Mass Index (kg/m²)				*P*-value
	Normal weight	Underweight	Overweight	Obesity	
	(*N* = 49)	(*N* = 939)	(*N* = 1,607)	(*N* = 635)	
BMI, (kg/m²)	21.89 ± 1.49	17.28 ± 1.14	25.97 ± 1.15	33.87 ± 5.22	< 0.001
Years since first cancer diagnosis, years	10.98 ± 11.02	14.05 ± 12.99	10.96 ± 11.07	11.88 ± 11.18	0.181
Cancer group, n (%)					0.002
Breast	104 (15.94%)	8 (16.22%)	138 (16.49%)	245 (14.80%)	
Prostate	73 (5.97%)	5 (4.38%)	171 (11.77%)	246 (9.76%)	
Non-melanoma/unspecified skin	159 (32.33%)	10 (28.05%)	224 (30.82%)	345 (28.22%)	
Colorectal	31 (3.85%)	3 (1.84%)	63 (5.33%)	108 (4.72%)	
Gynecologic	74 (12.91%)	9 (26.74%)	87 (9.51%)	246 (14.86%)	
Melanoma	46 (9.16%)	0 (0.00%)	45 (5.57%)	100 (7.79%)	
Hematologic	24 (3.37%)	3 (3.77%)	37 (3.63%)	59 (3.99%)	
Lung	23 (3.31%)	3 (5.18%)	14 (0.89%)	33 (1.59%)	
Urinary	20 (2.18%)	1 (1.62%)	48 (4.58%)	57 (2.51%)	
Other/unknown	81 (10.98%)	7 (12.20%)	112 (11.39%)	168 (11.75%)	
Age, n (%)					0.155
< 65	261 (51.96%)	19 (45.87%)	323 (46.78%)	717 (53.37%)	
≥ 65	374 (48.04%)	30 (54.13%)	616 (53.22%)	890 (46.63%)	
Sex, n (%)					< 0.001
Male	270 (33.98%)	16 (18.44%)	499 (47.02%)	745 (45.28%)	
Female	365 (66.02%)	33 (81.56%)	440 (52.98%)	862 (54.72%)	
Race/ethnicity, n (%)					0.058
Mexican American	24 (1.37%)	0 (0.00%)	54 (1.83%)	125 (2.93%)	
Non-Hispanic White	481 (89.17%)	35 (89.68%)	669 (86.87%)	1070 (85.76%)	
Non-Hispanic Black	68 (3.71%)	11 (6.47%)	120 (4.88%)	254 (5.95%)	
Other	62 (5.76%)	3 (3.85%)	96 (6.42%)	158 (5.36%)	
Education level, n (%)					0.633
< High school	46 (3.43%)	5 (6.93%)	93 (5.01%)	140 (4.19%)	
Completed high school	69 (8.37%)	6 (10.04%)	112 (7.69%)	204 (8.86%)	
> High school	520 (88.19%)	38 (83.03%)	734 (87.30%)	1263 (86.95%)	
PIR, n (%)					0.004
< 1.30	137 (12.77%)	17 (30.80%)	194 (12.07%)	417 (16.54%)	
1.30–3.49	254 (35.35%)	17 (29.24%)	395 (35.20%)	663 (38.05%)	
≥ 3.5	244 (51.88%)	15 (39.96%)	350 (52.73%)	527 (45.41%)	
Marital status, n (%)					0.089
Married/Living with partner	368 (62.02%)	27 (57.48%)	576 (66.17%)	997 (68.76%)	
Living alone	267 (37.98%)	22 (42.52%)	363 (33.83%)	610 (31.24%)	
Alcohol status, n (%)					0.104
No	207 (26.67%)	21 (34.15%)	387 (32.38%)	645 (32.80%)	
Yes	428 (73.33%)	28 (65.85%)	552 (67.62%)	962 (67.20%)	
Smoking status, n (%)					< 0.001
Never	274 (45.32%)	13 (35.36%)	424 (45.81%)	705 (44.25%)	
Former	219 (31.55%)	17 (29.91%)	379 (39.19%)	687 (41.96%)	
Current	142 (23.13%)	19 (34.73%)	136 (15.01%)	215 (13.79%)	
Hypertension, n (%)					< 0.001
No	303 (58.28%)	21 (48.20%)	359 (45.81%)	477 (33.08%)	
Yes	332 (41.72%)	28 (51.80%)	580 (54.19%)	1130 (66.92%)	
Diabetes mellitus, n (%)					< 0.001
No	561 (91.75%)	46 (95.00%)	762 (86.11%)	1062 (69.96%)	
Yes	74 (8.25%)	3 (5.00%)	177 (13.89%)	545 (30.04%)	
CVD history, n (%)					0.103
No	506 (85.28%)	37 (77.14%)	711 (81.54%)	1219 (79.96%)	
Yes	129 (14.72%)	12 (22.86%)	228 (18.46%)	388 (20.04%)	

Data are presented as survey-weighted means ± standard deviation or unweighted n (survey-weighted %)

P-values were calculated using survey-weighted linear regression for continuous variables and Rao–Scott chi-square tests for categorical variables

### Relationships between obesity indicators and mortality

Using the reverse Kaplan–Meier method, the survey-weighted median potential follow-up was 7.9 years (IQR, 4.3–12.1). A total of 937 all-cause, 315 cancer-specific, and 197 CVD deaths occurred. Weighted Kaplan-Meier curves showed separation across ABSI quartiles for all three outcomes (all *P* < 0.0001). BRI quartiles differed for all-cause (*P* = 0.002) and CVD mortality (*P* = 0.001) but not cancer mortality (*P* = 0.673). No statistically significant differences were observed across BMI categories (all-cause *P* = 0.092, cancer-specific *P* = 0.607, and CVD *P* = 0.885; Figure S2).

**Fig. 1:**
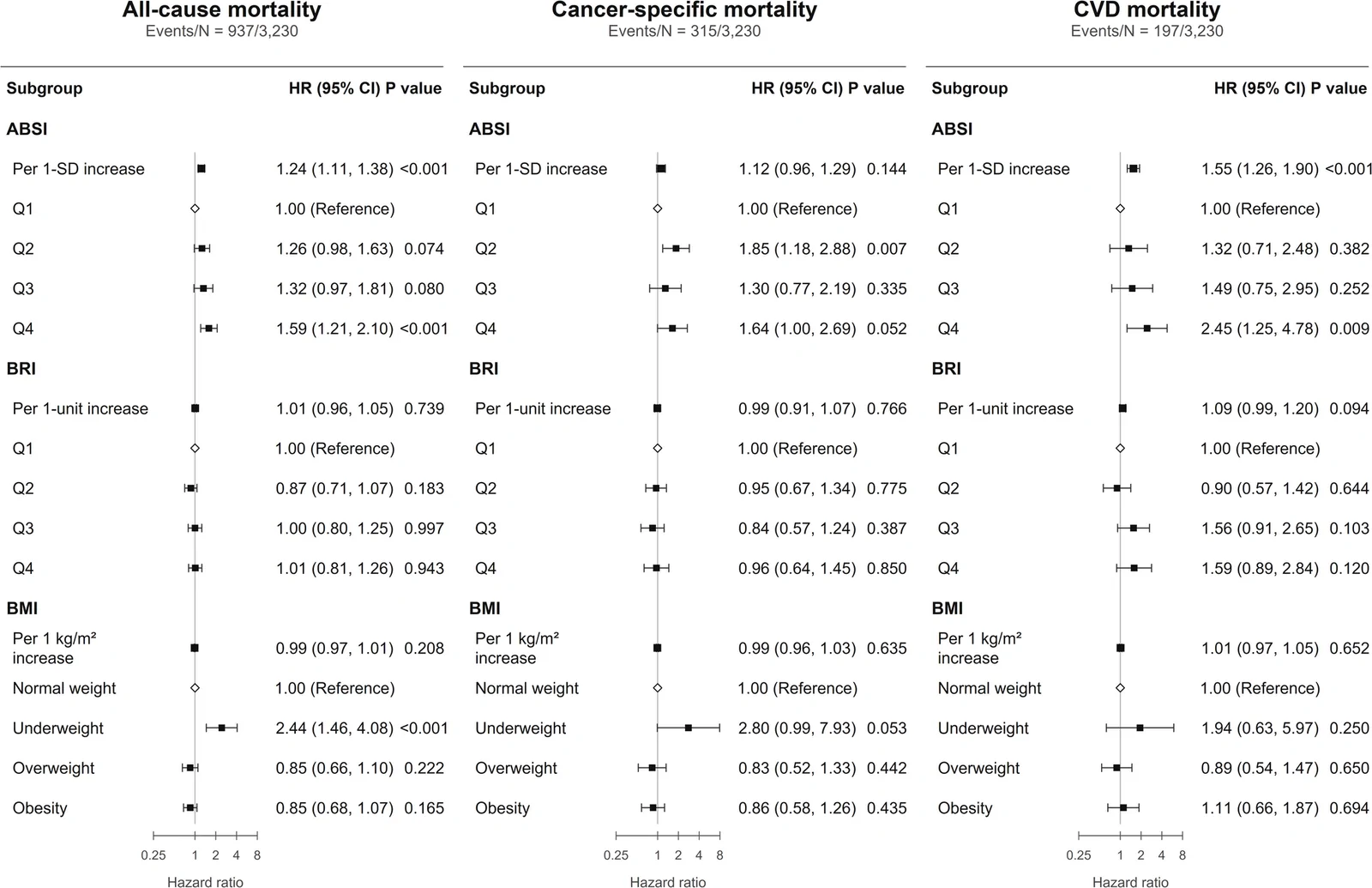
Forest plot showing the adjusted associations of ABSI, BRI, and BMI with all-cause, cancer-specific, and CVD mortality among adults with a self-reported history of cancer in Model 3. ABSI is scaled per 1 SD; BRI per 1 unit; BMI per 1 kg/m². Model 3 adjusts for Age, Sex, Race/ethnicity, Education level, PIR, Marital status, Alcohol status, Smoking status, cancer group, and years since first cancer diagnosis

Three sets of weighted Cox regression models were constructed to explore the independent associations between the obesity indices and all-cause mortality, cancer-specific mortality and CVD mortality (Fig. 1, Table S3). In Model 3, each 1-SD higher ABSI was associated with higher all-cause mortality (HR 1.24, 95% CI 1.11–1.38; *P* < 0.001) and CVD mortality (HR 1.55, 95% CI 1.26–1.90; *P* < 0.001), but not cancer mortality (HR 1.12, 95% CI 0.96–1.29; *P* = 0.144). Compared with Q1, ABSI Q4 was associated with all-cause (HR 1.59, 95% CI 1.21–2.10; *P* < 0.001) and CVD mortality (HR 2.45, 95% CI 1.25–4.78; *P* = 0.009); the Q4 cancer estimate was borderline (HR 1.64, 95% CI 1.00-2.69; *P* = 0.052).

In contrast, BRI was not significantly associated with any mortality outcome in Model 3. Similarly, no significant trends were observed across BRI quartiles for all-cause, cancer, or CVD mortality. Continuous BMI was also not associated with these outcomes. Underweight, however, was associated with higher all-cause mortality versus normal weight (HR 2.44, 95% CI 1.46–4.08; *P* < 0.001).

### Smooth curve fitting

**Fig. 2:**
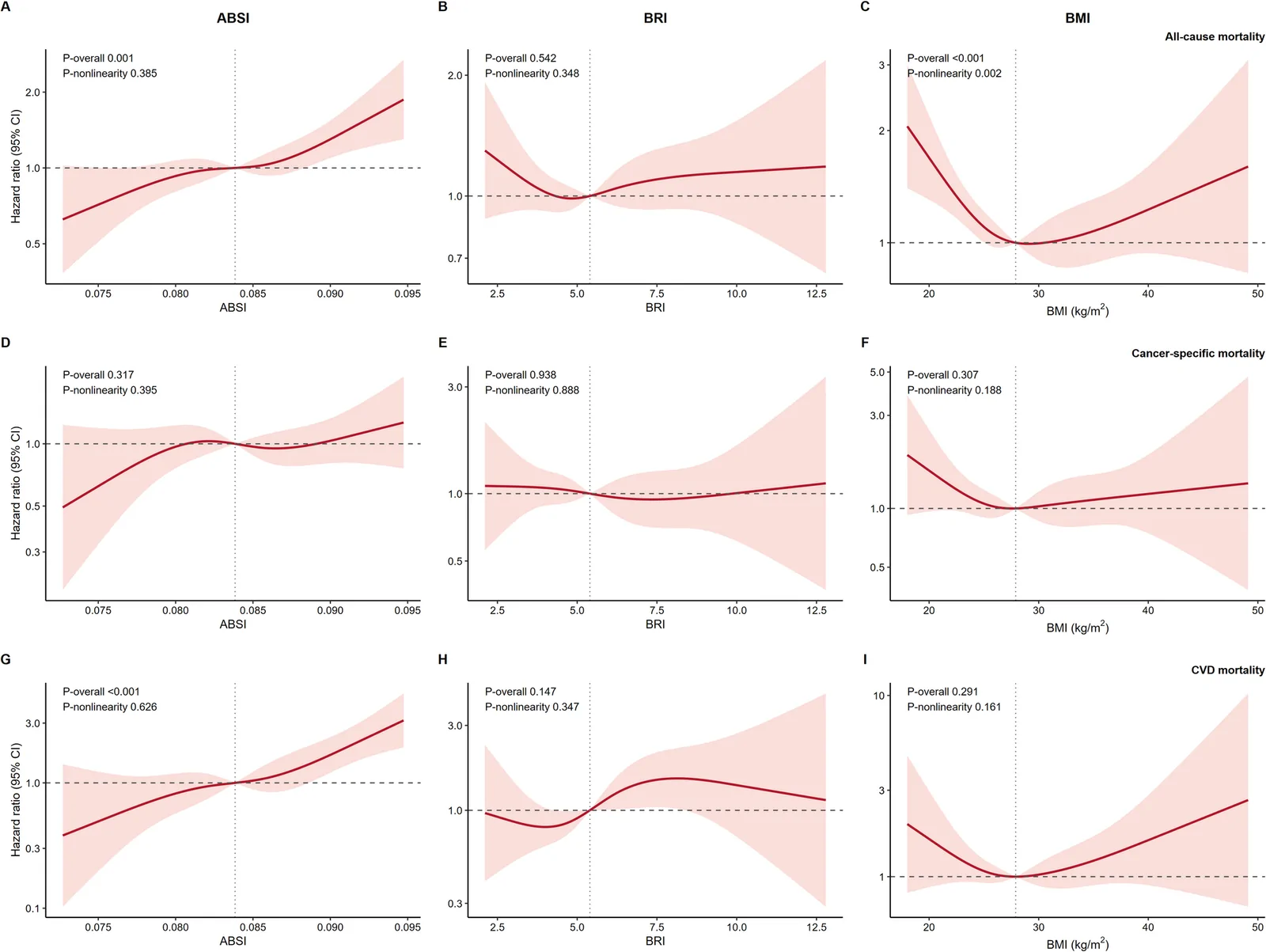
Survey-weighted restricted cubic spline associations of ABSI, BRI, and BMI with mortality in Model 3. Panels (A–C) show the associations of ABSI, BRI, and BMI, respectively, with all-cause mortality; panels (D–F) show the corresponding associations with cancer-specific mortality; and panels (G–I) show the associations with CVD mortality. Knots were placed at the survey-weighted 5th, 35th, 65th, and 95th percentiles; the survey-weighted median was used as the reference. Solid red lines represent hazard ratios (HRs) on a logarithmic scale, and shaded areas indicate 95% confidence intervals (CIs). Vertical dotted lines indicate the median reference values

RCS analyses supported overall associations of ABSI with all-cause (*P* = 0.001) and CVD mortality (*P* < 0.001), without evidence of nonlinearity (*P* = 0.385 and *P* = 0.626, respectively). BMI had a nonlinear association with all-cause mortality (P overall < 0.001; P nonlinearity = 0.002), characterized by a higher all-cause mortality hazard at the lower end of the BMI distribution. No nonlinear association was detected for BMI with CVD mortality or for BRI with any outcome (Fig. 2; Table S8).

### Sensitivity and subgroup analyses

The approximate residual-based diagnostics did not indicate exposure-specific nonproportionality in any of the nine primary Cox models (diagnostic *P* = 0.117–0.956). However, some global diagnostics suggested possible departures from proportional hazards. After stratifying the baseline hazard by covariates flagged in the initial diagnostics, no residual global evidence of nonproportionality was detected, although these diagnostics remained approximate and exploratory. The exposure estimates remained similar to those from Model 3: the ABSI HR was 1.22 (95% CI 1.09–1.36) for all-cause mortality and 1.55 (95% CI 1.25–1.91) for CVD mortality (Tables S5A–B).

The 12-month landmark analysis included 3,137 participants, with 854 all-cause, 270 cancer-specific, and 183 CVD deaths. The corresponding 24-month analysis included 2,841 participants, with 757, 221, and 166 deaths, respectively. The estimated associations were generally similar to those in the primary analysis (Table S6**)**. At the 24-month landmark, the ABSI HRs were 1.25 (95% CI 1.11–1.41) for all-cause mortality and 1.56 (95% CI 1.24–1.96) for CVD mortality. In the approximate survey-weighted Fine–Gray sensitivity analysis, each 1-SD higher ABSI was associated with CVD mortality (sHR 1.46, 95% CI 1.21–1.76), whereas no statistically significant association was observed with cancer-specific mortality (Table S7). In sensitivity analysis Model 4 (Table S4), ABSI remained associated with all-cause (HR 1.19, 95% CI 1.07–1.32) and CVD mortality (HR 1.43, 95% CI 1.16–1.76).

**Fig. 3:**
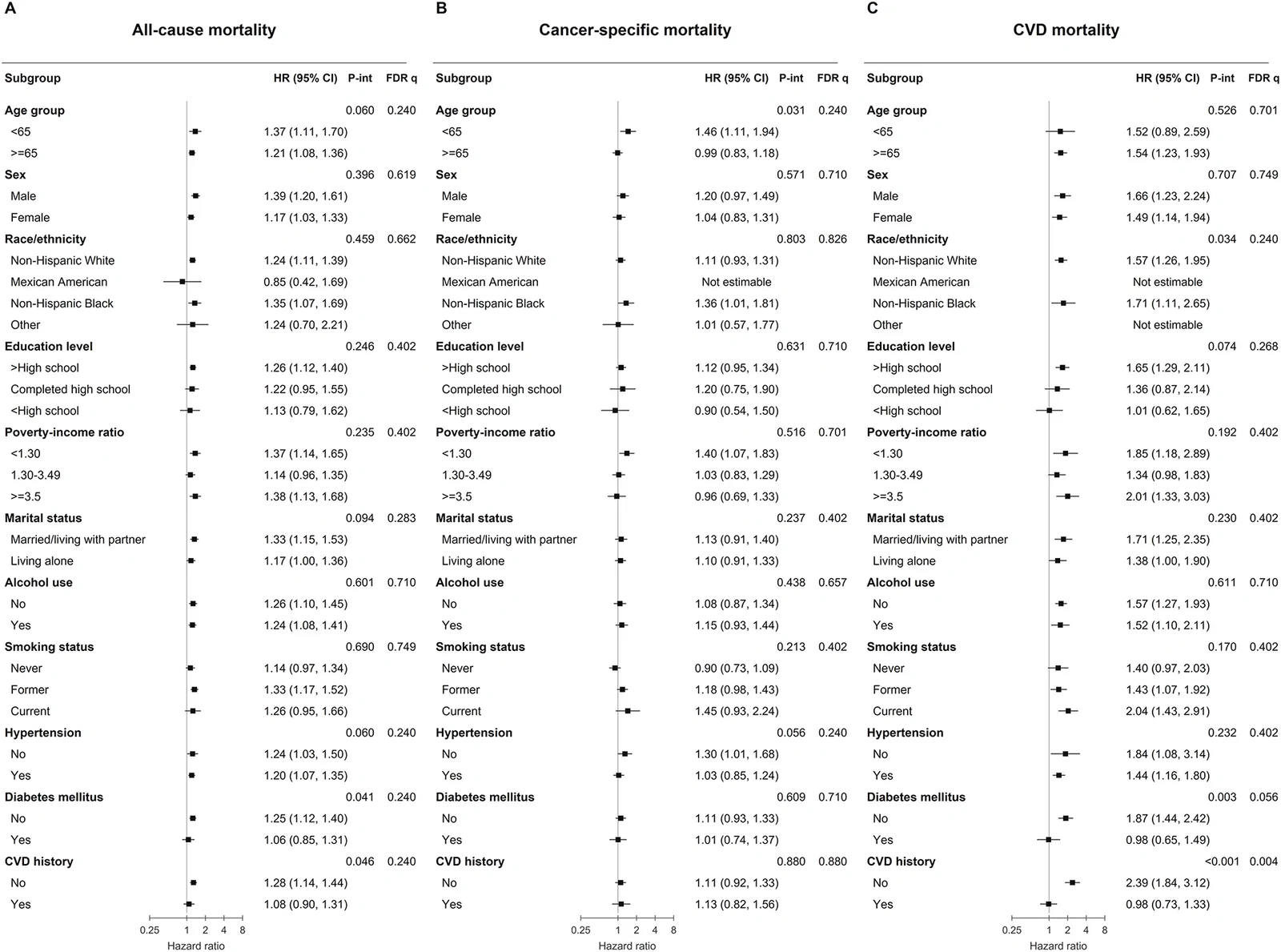
Exploratory survey-weighted subgroup analyses of the association per 1-SD higher ABSI with mortality. Panels (A), (B), and (C) present the subgroup analyses for all-cause mortality, cancer-specific mortality, and cardiovascular disease (CVD) mortality, respectively. Within-subgroup models used the Model 3 covariates except the stratifying variable. P values for interaction and Benjamini–Hochberg q values calculated across 36 ABSI subgroup interaction tests are shown; the correction includes the cancer-group interactions presented separately in Figure S5. Sparse strata with singular models are labeled not estimable

Exploratory ABSI interaction tests produced several nominal P values < 0.05, but after FDR correction only modification of the ABSI-CVD mortality association by baseline CVD history remained statistically significant (q = 0.004). No ABSI interaction for all-cause or cancer mortality survived correction (Fig. 3; Table S9). Additional cancer-group analyses provided no clear evidence that the associations of ABSI with all-cause, cancer-specific, or CVD mortality differed across cancer groups (interaction *P* = 0.083, 0.193, and 0.120; corresponding q = 0.273, 0.402, and 0.331 across the 36 ABSI subgroup tests; Table S9A, Table S12A, and Figure S5). An exploratory interaction between BRI and cancer group was observed for cancer-specific mortality (*P* = 0.000224; q = 0.002 across nine cancer-group tests), whereas the corresponding BMI interaction did not meet the 0.05 false-discovery-rate threshold (*P* = 0.012; q = 0.053; Tables S12B–S12C and S13).

After excluding participants reporting non-melanoma or unspecified skin cancer, 2,492 participants remained, including 730 all-cause, 266 cancer-specific, and 151 CVD deaths. The Model 3 hazard ratios per one-unit higher pooled ABSI z score were 1.22 (95% CI 1.10–1.34) for all-cause mortality, 1.09 (0.92–1.30) for cancer-specific mortality, and 1.50 (1.21–1.87) for CVD mortality (Table S14). These estimates were consistent in direction with the main analysis.

The covariate-only multiple-imputation analysis included 3,714 participants and 1,079 all-cause, 358 cancer-specific, and 228 CVD deaths. In Model 3, each 1-SD higher ABSI was associated with all-cause mortality (HR 1.25, 95% CI 1.14–1.38; *P* < 0.001) and CVD mortality (HR 1.47, 95% CI 1.22–1.78; *P* < 0.001), but not cancer-specific mortality (HR 1.11, 95% CI 0.96–1.28; *P* = 0.160). Continuous BRI and BMI were not significantly associated with any mortality outcome. Across Models 3 and 4, all 18 continuous-exposure estimates from the multiple-imputation analysis showed the same direction of association as the corresponding complete-case estimates (Table S11; Figure S4).

## Discussion

In this survey-weighted study of 3,230 U.S. adults with a self-reported history of cancer, a higher ABSI was associated with higher all-cause and CVD mortality after adjustment for sociodemographic, behavioral, and cancer-related characteristics. Specifically, each 1-SD higher ABSI was associated with a 24% higher all-cause mortality hazard and a 55% higher CVD mortality hazard in the primary model. In contrast, continuous ABSI was not significantly associated with cancer-specific mortality. Continuous BRI and BMI were not independently associated with any of the three mortality outcomes in the primary model. However, underweight BMI was associated with higher all-cause mortality, and the spline analysis showed a nonlinear association between BMI and all-cause mortality, primarily characterized by higher mortality at lower BMI values. These findings describe epidemiologic associations and should not be interpreted as demonstrating causal effects, predictive superiority, or clinical utility.

ABSI was developed to characterize waist circumference relative to BMI and height and may capture aspects of body shape that are not fully reflected by BMI alone [7]. Previous studies have associated higher ABSI with mortality in general populations [7, 25] and with the incidence of several cancers [8]. ABSI has also been examined in relation to prostate cancer-specific mortality; however, the reported positive association was primarily observed among men diagnosed within four years rather than in the overall analytical population [16]. Our findings extend this evidence to a heterogeneous population of adults with a self-reported cancer history while additionally accounting for cancer group and years since first cancer diagnosis. Continuous ABSI was not significantly associated with cancer-specific mortality in the present analysis. However, the categorical analysis showed higher cancer-specific mortality in Q2 than in Q1 (HR 1.85, 95% CI 1.18–2.88), without a consistent monotonic pattern across quartiles. This isolated categorical finding warrants cautious interpretation, particularly given the multiple comparisons, and does not establish a clear dose–response relationship. Differences in cancer composition, stage, treatment, follow-up duration, and the number of cause-specific deaths may partly explain the variation between studies. Although ABSI showed more consistent associations than BRI or BMI in the present models, we did not evaluate discrimination, calibration, reclassification, or external prediction performance.

Cardiometabolic pathways may provide one possible explanation for the observed associations of ABSI with all-cause and CVD mortality. Abdominal adiposity has been associated with insulin resistance, adipose-tissue inflammation, systemic metabolic dysregulation, and adverse cardiometabolic profiles [26]. ABSI may capture some aspects of abdominal body shape that are not adequately reflected by BMI. However, ABSI and BRI are derived from routine anthropometric measurements and do not directly quantify visceral adipose tissue, ectopic fat, or skeletal muscle. Direct measurements of body composition and mechanistic biomarkers were not incorporated into these analyses, and no formal mediation analysis was performed. The present study therefore cannot determine whether visceral adiposity or any specific biological pathway mediated the observed associations.

For BRI, weighted Kaplan-Meier analyses showed differences in the unadjusted survival distributions across quartiles for all-cause and CVD mortality. Several associations were also present in the unadjusted Cox models, but they were attenuated after covariate adjustment, and neither continuous nor categorical BRI was independently associated with mortality in the primary model. The attenuation after covariate adjustment suggests that differences in participant characteristics may partly account for the unadjusted survival differences across BRI quartiles. Survey weighting accounts for the sampling design but does not, by itself, adjust for confounding. Although a positive BRI-CVD mortality association appeared in the 24-month landmark analysis, it was not consistently observed in the primary, extended-adjustment, spline, or competing-risk analyses and should therefore be considered hypothesis-generating. Differences from previous general-population BRI studies [10, 27] may reflect variation in study populations, outcome definitions, exposure parameterization, and covariate-adjustment strategies.

The BMI findings also require cautious interpretation. The underweight group included only 49 participants, limiting the precision of the estimates, particularly for cause-specific mortality. Underweight participants had higher all-cause mortality, and the spline analysis indicated greater all-cause mortality at the lower end of the BMI distribution. Low BMI among adults with a history of cancer may reflect frailty, reduced skeletal muscle mass, cancer-associated cachexia, disease severity, treatment-related weight loss, or other underlying illnesses rather than a harmful effect of low adiposity itself [28, 29]. Persistence after the 12- and 24-month landmark exclusions suggests that early deaths did not fully explain the association, but these analyses cannot eliminate reverse causation because weight loss and body-composition changes may precede death by several years. Neither overweight nor obesity was significantly associated with lower all-cause mortality in the primary model. The inverse BMI associations observed in Model 4 should not be interpreted as evidence that obesity is protective. These estimates were model-dependent and may have been affected by adjustment for hypertension, diabetes, and CVD history, which could partly lie on the pathway between adiposity and mortality; adjustment for intermediate variables may produce overadjustment bias when estimating the total association [20]. Furthermore, restricting the study population to individuals with a cancer history who survived until the NHANES examination may have introduced survivor-selection or collider-stratification bias [30].

The main ABSI findings were generally consistent across complementary analyses. Associations with all-cause and CVD mortality persisted, although somewhat attenuated, after additional cardiometabolic adjustment in Model 4. Similar estimates were obtained in the proportional-hazards-stratified models and the 12- and 24-month landmark analyses. The association with CVD mortality also remained in the Fine-Gray competing-risk analysis, whereas no association with cancer-specific mortality was detected. Results were materially unchanged in the covariate-only multiple-imputation analysis, which retained 484 participants excluded from the complete-case analysis and reduced concern that missing covariate data drove the primary findings. Nevertheless, Model 4 should be interpreted as an extended cardiometabolic sensitivity model rather than as a model estimating a controlled direct effect [20]. Multiple imputation also relies on assumptions regarding the mechanism of missingness. Consistency across these analyses supports robustness to several modeling choices but does not remove residual confounding or establish causality.

Among the ABSI subgroup interaction tests, only the interaction between ABSI and baseline CVD history for CVD mortality remained statistically significant after false-discovery-rate correction. The estimated ABSI–CVD mortality association was stronger among participants without reported baseline CVD than among those with a CVD history, consistent with this adjusted interaction test. Differences in cardiovascular treatment and risk-factor management may partly contribute to this pattern, although these explanations could not be directly evaluated in the present study [31]. Selection related to prevalent disease and survival to study entry may also influence the observed associations [32]. This finding requires confirmation in independent cohorts and should not currently be used for clinical risk stratification [33].

Cancer-group analyses provide additional context for the heterogeneity of this survivor population [34]. The absence of statistically significant ABSI interactions should not be interpreted as equivalence across cancer types, given the limited number of cause-specific deaths in several groups [35]. The absence of an overall BRI association does not preclude variation in associations across cancer groups, because the overall model estimates a common association whereas the interaction model evaluates between-group heterogeneity. Nevertheless, the BRI–cancer mortality interaction was identified in a post hoc analysis and remains hypothesis-generating. Its interpretation depends on the assumed linear exposure effects and common covariate coefficients, and estimates in cancer groups with few deaths require particular caution (Table S13). The persistence of the ABSI associations with all-cause and CVD mortality after excluding non-melanoma or unspecified skin cancer reduces concern that this relatively large group alone explains the overall findings (Table S14). However, these analyses cannot eliminate residual confounding by unmeasured cancer severity or treatment. Selection and disease heterogeneity may also influence associations between adiposity and survival after cancer diagnosis [36].

## Advantages and limitations

The strengths of this study include the use of a large nationally representative survey with standardized anthropometric measurements, linked mortality follow-up, and analyses accounting for the NHANES complex sampling design. ABSI, BRI, and BMI were evaluated within the same analytical population and adjustment framework. Cancer group and years since first cancer diagnosis were incorporated into the primary model, and the robustness of the findings was examined using nonlinear models, proportional-hazards diagnostics, landmark analyses, competing-risk models, alternative cardiometabolic adjustment, correction for multiple subgroup tests, and covariate-only multiple imputation.

Several limitations should also be considered. First, this was an observational study based on anthropometric measurements obtained at a single examination; therefore, temporal changes in body composition could not be assessed, and causal or predictive conclusions cannot be drawn. Second, cancer history and age at diagnosis were self-reported. Although cancer group and years since first diagnosis were included, information on cancer stage, grade, recurrence, treatment, treatment intent, response, and treatment-related weight change was unavailable. Considerable heterogeneity consequently remained within the cancer groups, and residual confounding by cancer severity and treatment is likely. Third, BMI, ABSI, and BRI cannot distinguish adipose tissue from skeletal muscle or directly measure visceral fat. Fourth, reverse causation from preclinical disease, frailty, cachexia, and illness-related weight loss cannot be completely excluded despite the landmark analyses. Restricting the study population to individuals who survived until the NHANES examination may also have introduced survivor-selection bias.

Fifth, the numbers of cancer-specific and CVD deaths were smaller than the number of all-cause deaths, limiting statistical precision, particularly in subgroup analyses. Finally, the primary analysis was restricted to complete cases. Although multiple-imputation analyses produced similar conclusions, these analyses rely on assumptions regarding the missing-data mechanism, and participants with missing anthropometric exposures or unusable mortality information were not recovered by imputation. The findings are therefore most directly generalizable to non-institutionalized U.S. adults with a self-reported history of cancer who were eligible for the NHANES examination and mortality linkage.

## Conclusion

Among U.S. adults with a self-reported history of cancer, higher ABSI was associated with higher all-cause and CVD mortality. No statistically significant association was observed between continuous ABSI and cancer-specific mortality. Continuous BRI and BMI were not significantly associated with mortality in the primary model, whereas underweight BMI was associated with higher all-cause mortality. These findings warrant further investigation in independent cohorts with detailed cancer characteristics and longitudinal body-composition measurements.

## Supplementary Information

Below is the link to the electronic supplementary material.

Supplementary Material 1

Supplementary Material 2

Supplementary Material 3

Supplementary Material 4

Supplementary Material 5

Supplementary Material 6

## Data Availability

The data used in this study are publicly available from the National Center for Health Statistics, including the National Health and Nutrition Examination Survey (NHANES; https://wwwn.cdc.gov/nchs/nhanes/) and the public-use Linked Mortality Files ( https://www.cdc.gov/nchs/linked-data/mortality-files/index.html).

## Abbreviations

ABSI: A body shape index
BMI: Body mass index
BRI: Body roundness index
CI: Confidence interval
CVD: Cardiovascular disease
FDR: False discovery rate
HR: Hazard ratio
MEC: Mobile examination center
NCHS: National Center for Health Statistics
NHANES: National Health and Nutrition Examination Survey
PIR: Poverty-to-income ratio
RCS: Restricted cubic spline
SD: Standard deviation
