# Supplementary material for: Associations of anthropometric indices with all-cause and cause-specific mortality among US adults with a self-reported history of cancer: a population-based cohort study
Source: BMC Endocr Disord. 2026 Oct 15;26:342. doi: 10.1186/s12902-026-02652-w (PMC13725999; doi:10.1186/s12902-026-02652-w)
Supplement: Supplementary file 1 [file 12902_2026_2652_MOESM6_ESM.docx]

**Table S1.** Survey-weighted baseline characteristics by ABSI quartile (N=3,230).

| Characteristic | A Body Shape Index (ABSI) | | | | P-value |
| --- | --- | --- | --- | --- | --- |
|  | Q1 (N=808) | Q2 (N=807) | Q3 (N=807) | Q4 (N=808) |  |
| Years since first cancer diagnosis, years | 10.80 ± 10.49 | 11.17 ± 10.64 | 12.18 ± 11.86 | 11.88 ± 11.72 | 0.119 |
| Cancer group, n (%) |  |  |  |  | <0.001 |
| Breast | 178 (20.93%) | 115 (14.11%) | 97 (10.82%) | 105 (15.38%) |  |
| Prostate | 50 (2.99%) | 127 (8.84%) | 157 (13.77%) | 161 (14.04%) |  |
| Non-melanoma/unspecified skin | 148 (27.04%) | 182 (30.78%) | 203 (31.96%) | 205 (29.90%) |  |
| Colorectal | 46 (3.81%) | 51 (5.33%) | 47 (3.69%) | 61 (6.14%) |  |
| Gynecologic | 174 (20.12%) | 110 (12.05%) | 85 (11.85%) | 47 (6.38%) |  |
| Melanoma | 45 (7.30%) | 49 (7.77%) | 46 (7.07%) | 51 (7.21%) |  |
| Hematologic | 33 (3.87%) | 35 (4.40%) | 26 (3.44%) | 29 (3.18%) |  |
| Lung | 11 (0.89%) | 10 (1.09%) | 22 (2.16%) | 30 (3.48%) |  |
| Urinary | 19 (1.34%) | 30 (3.18%) | 29 (2.98%) | 48 (5.11%) |  |
| Other/unknown | 104 (11.71%) | 98 (12.46%) | 95 (12.27%) | 71 (9.16%) |  |
| Age, n (%) |  |  |  |  | <0.001 |
| <65 | 541 (72.25%) | 377 (56.67%) | 262 (43.97%) | 140 (24.05%) |  |
| ≥65 | 267 (27.75%) | 430 (43.33%) | 545 (56.03%) | 668 (75.95%) |  |
| Sex, n (%) |  |  |  |  | <0.001 |
| Male | 192 (22.55%) | 374 (44.46%) | 473 (55.36%) | 491 (54.86%) |  |
| Female | 616 (77.45%) | 433 (55.54%) | 334 (44.64%) | 317 (45.14%) |  |
| Race/ethnicity, n (%) |  |  |  |  | <0.001 |
| Mexican American | 56 (2.53%) | 62 (2.50%) | 52 (2.45%) | 33 (1.36%) |  |
| Non-Hispanic White | 482 (82.12%) | 542 (87.68%) | 575 (87.42%) | 656 (91.51%) |  |
| Non-Hispanic Black | 171 (7.97%) | 118 (4.88%) | 96 (4.26%) | 68 (2.86%) |  |
| Other | 99 (7.38%) | 85 (4.93%) | 84 (5.87%) | 51 (4.27%) |  |
| Education level, n (%) |  |  |  |  | <0.001 |
| <High school | 43 (2.19%) | 70 (3.86%) | 76 (5.03%) | 95 (6.85%) |  |
| Completed high school | 77 (6.81%) | 98 (7.95%) | 98 (7.42%) | 118 (12.39%) |  |
| >High school | 688 (90.99%) | 639 (88.19%) | 633 (87.55%) | 595 (80.76%) |  |
| PIR, n (%) |  |  |  |  | <0.001 |
| <1.30 | 189 (14.04%) | 194 (15.21%) | 187 (14.72%) | 195 (14.89%) |  |
| 1.30-3.49 | 277 (29.49%) | 320 (33.02%) | 338 (37.29%) | 394 (49.54%) |  |
| ≥3.5 | 342 (56.47%) | 293 (51.77%) | 282 (47.99%) | 219 (35.57%) |  |
| Marital status, n (%) |  |  |  |  | 0.054 |
| Married/Living with partner | 471 (65.69%) | 502 (69.00%) | 516 (68.96%) | 479 (61.60%) |  |
| Living alone | 337 (34.31%) | 305 (31.00%) | 291 (31.04%) | 329 (38.40%) |  |
| Alcohol status, n (%) |  |  |  |  | <0.001 |
| No | 280 (26.04%) | 296 (29.43%) | 317 (32.22%) | 367 (40.16%) |  |
| Yes | 528 (73.96%) | 511 (70.57%) | 490 (67.78%) | 441 (59.84%) |  |
| Smoking status, n (%) |  |  |  |  | <0.001 |
| Never | 453 (57.68%) | 346 (40.97%) | 328 (41.15%) | 289 (36.25%) |  |
| Former | 221 (26.79%) | 318 (39.92%) | 363 (43.85%) | 400 (48.02%) |  |
| Current | 134 (15.53%) | 143 (19.11%) | 116 (15.00%) | 119 (15.73%) |  |
| Hypertension, n (%) |  |  |  |  | <0.001 |
| No | 394 (54.55%) | 302 (46.34%) | 259 (36.22%) | 205 (27.07%) |  |
| Yes | 414 (45.45%) | 505 (53.66%) | 548 (63.78%) | 603 (72.93%) |  |
| Diabetes mellitus, n (%) |  |  |  |  | <0.001 |
| No | 683 (88.37%) | 623 (81.19%) | 584 (77.39%) | 541 (67.51%) |  |
| Yes | 125 (11.63%) | 184 (18.81%) | 223 (22.61%) | 267 (32.49%) |  |
| CVD history, n (%) |  |  |  |  | <0.001 |
| No | 710 (91.00%) | 632 (83.49%) | 593 (78.57%) | 538 (69.55%) |  |
| Yes | 98 (9.00%) | 175 (16.51%) | 214 (21.43%) | 270 (30.45%) |  |

*Data are presented as survey-weighted means ± standard deviation or unweighted n (survey-weighted %).*

*P-values were calculated using survey-weighted linear regression for continuous variables and Rao–Scott chi-square tests for categorical variables.*

*NHANES complex survey design variables SDMVSTRA and SDMVPSU and the 16-year MEC weight WTMEC16YR were incorporated.*

**Table S2.** Survey-weighted baseline characteristics by BRI quartile (N=3,230).

| Characteristic | Body Roundness Index (BRI) | | | | P-value |
| --- | --- | --- | --- | --- | --- |
|  | Q1 (N=808) | Q2 (N=807) | Q3 (N=807) | Q4 (N=808) |  |
| Years since first cancer diagnosis, years | 10.74 ± 11.15 | 11.29 ± 10.81 | 12.21 ± 11.65 | 11.77 ± 10.96 | 0.164 |
| Cancer group, n (%) |  |  |  |  | <0.001 |
| Breast | 127 (16.79%) | 99 (11.98%) | 120 (14.66%) | 149 (18.70%) |  |
| Prostate | 103 (6.12%) | 147 (12.09%) | 138 (10.36%) | 107 (9.74%) |  |
| Non-melanoma/unspecified skin | 209 (34.83%) | 184 (28.80%) | 190 (30.26%) | 155 (24.51%) |  |
| Colorectal | 34 (3.40%) | 56 (5.47%) | 54 (4.59%) | 61 (5.40%) |  |
| Gynecologic | 108 (14.33%) | 74 (8.91%) | 96 (12.58%) | 138 (16.65%) |  |
| Melanoma | 42 (6.19%) | 50 (8.62%) | 59 (9.06%) | 40 (5.68%) |  |
| Hematologic | 37 (3.76%) | 31 (3.99%) | 31 (4.06%) | 24 (3.20%) |  |
| Lung | 22 (2.11%) | 16 (1.49%) | 18 (1.88%) | 17 (1.69%) |  |
| Urinary | 25 (2.31%) | 36 (3.97%) | 26 (2.33%) | 39 (3.50%) |  |
| Other/unknown | 101 (10.15%) | 114 (14.70%) | 75 (10.21%) | 78 (10.93%) |  |
| Age, n (%) |  |  |  |  | <0.001 |
| <65 | 406 (59.71%) | 297 (49.95%) | 280 (44.12%) | 337 (49.04%) |  |
| ≥65 | 402 (40.29%) | 510 (50.05%) | 527 (55.88%) | 471 (50.96%) |  |
| Sex, n (%) |  |  |  |  | <0.001 |
| Male | 365 (37.57%) | 437 (49.54%) | 414 (49.13%) | 314 (36.61%) |  |
| Female | 443 (62.43%) | 370 (50.46%) | 393 (50.87%) | 494 (63.39%) |  |
| Race/ethnicity, n (%) |  |  |  |  | 0.014 |
| Mexican American | 27 (1.22%) | 39 (1.50%) | 63 (3.35%) | 74 (3.21%) |  |
| Non-Hispanic White | 577 (87.63%) | 583 (87.48%) | 565 (86.76%) | 530 (85.30%) |  |
| Non-Hispanic Black | 124 (5.19%) | 103 (4.55%) | 108 (5.23%) | 118 (5.82%) |  |
| Other | 80 (5.96%) | 82 (6.48%) | 71 (4.66%) | 86 (5.67%) |  |
| Education level, n (%) |  |  |  |  | <0.001 |
| <High school | 46 (2.34%) | 72 (4.55%) | 87 (6.27%) | 79 (4.42%) |  |
| Completed high school | 80 (7.07%) | 82 (6.78%) | 104 (9.89%) | 125 (10.41%) |  |
| >High school | 682 (90.59%) | 653 (88.66%) | 616 (83.84%) | 604 (85.17%) |  |
| PIR, n (%) |  |  |  |  | <0.001 |
| <1.30 | 168 (12.18%) | 155 (11.61%) | 203 (15.62%) | 239 (20.00%) |  |
| 1.30-3.49 | 286 (28.87%) | 351 (38.34%) | 349 (39.94%) | 343 (40.43%) |  |
| ≥3.5 | 354 (58.95%) | 301 (50.05%) | 255 (44.43%) | 226 (39.57%) |  |
| Marital status, n (%) |  |  |  |  | 0.051 |
| Married/Living with partner | 488 (64.01%) | 534 (71.95%) | 476 (64.99%) | 470 (64.98%) |  |
| Living alone | 320 (35.99%) | 273 (28.05%) | 331 (35.01%) | 338 (35.02%) |  |
| Alcohol status, n (%) |  |  |  |  | <0.001 |
| No | 248 (23.99%) | 319 (32.97%) | 333 (32.32%) | 360 (37.66%) |  |
| Yes | 560 (76.01%) | 488 (67.03%) | 474 (67.68%) | 448 (62.34%) |  |
| Smoking status, n (%) |  |  |  |  | <0.001 |
| Never | 360 (47.80%) | 362 (44.94%) | 330 (39.66%) | 364 (46.10%) |  |
| Former | 270 (31.02%) | 319 (38.32%) | 365 (45.62%) | 348 (41.97%) |  |
| Current | 178 (21.17%) | 126 (16.74%) | 112 (14.72%) | 96 (11.93%) |  |
| Hypertension, n (%) |  |  |  |  | <0.001 |
| No | 429 (62.65%) | 306 (43.94%) | 212 (28.59%) | 213 (29.29%) |  |
| Yes | 379 (37.35%) | 501 (56.06%) | 595 (71.41%) | 595 (70.71%) |  |
| Diabetes mellitus, n (%) |  |  |  |  | <0.001 |
| No | 729 (93.46%) | 670 (86.76%) | 567 (73.82%) | 465 (60.48%) |  |
| Yes | 79 (6.54%) | 137 (13.24%) | 240 (26.18%) | 343 (39.52%) |  |
| CVD history, n (%) |  |  |  |  | <0.001 |
| No | 664 (87.71%) | 636 (83.35%) | 579 (76.24%) | 594 (77.26%) |  |
| Yes | 144 (12.29%) | 171 (16.65%) | 228 (23.76%) | 214 (22.74%) |  |

*Data are presented as survey-weighted means ± standard deviation or unweighted n (survey-weighted %).*

*P-values were calculated using survey-weighted linear regression for continuous variables and Rao–Scott chi-square tests for categorical variables.*

*NHANES complex survey design variables SDMVSTRA and SDMVPSU and the 16-year MEC weight WTMEC16YR were incorporated.*

**Table S3.** Survey-weighted Cox models for obesity indices and mortality (Models 1-3; N=3,230).

| **All-cause mortality (937/3,230)** | | | | | | |
| --- | --- | --- | --- | --- | --- | --- |
| Variables | Model 1 |  | Model 2 |  | Model 3 | P value |
|  | HR (95% CI) | P value | HR (95% CI) | P value | HR (95% CI) |  |
| **ABSI** |  |  |  |  |  |  |
| Per 1-SD increase | 1.79 (1.64, 1.97) | <0.001 | 1.38 (1.24, 1.53) | <0.001 | 1.24 (1.11, 1.38) | <0.001 |
| Q1 | 1.00 (Reference) |  | 1.00 (Reference) |  | 1.00 (Reference) |  |
| Q2 | 1.83 (1.43, 2.35) | <0.001 | 1.39 (1.08, 1.79) | 0.010 | 1.26 (0.98, 1.63) | 0.074 |
| Q3 | 2.53 (1.88, 3.40) | <0.001 | 1.53 (1.13, 2.09) | 0.007 | 1.32 (0.97, 1.81) | 0.080 |
| Q4 | 4.27 (3.33, 5.49) | <0.001 | 2.07 (1.56, 2.73) | <0.001 | 1.59 (1.21, 2.10) | <0.001 |
| **BRI** |  |  |  |  |  |  |
| Per 1-unit increase | 1.05 (1.01, 1.09) | 0.011 | 1.03 (0.98, 1.08) | 0.230 | 1.01 (0.96, 1.05) | 0.739 |
| Q1 | 1.00 (Reference) |  | 1.00 (Reference) |  | 1.00 (Reference) |  |
| Q2 | 1.18 (0.95, 1.47) | 0.125 | 0.94 (0.77, 1.14) | 0.501 | 0.87 (0.71, 1.07) | 0.183 |
| Q3 | 1.51 (1.21, 1.89) | <0.001 | 1.08 (0.85, 1.36) | 0.533 | 1.00 (0.80, 1.25) | 0.997 |
| Q4 | 1.40 (1.12, 1.75) | 0.003 | 1.14 (0.90, 1.45) | 0.266 | 1.01 (0.81, 1.26) | 0.943 |
| **BMI** |  |  |  |  |  |  |
| Per 1 kg/m² increase | 0.98 (0.97, 1.00) | 0.052 | 0.99 (0.97, 1.01) | 0.215 | 0.99 (0.97, 1.01) | 0.208 |
| Normal weight | 1.00 (Reference) |  | 1.00 (Reference) |  | 1.00 (Reference) |  |
| Underweight | 2.15 (1.17, 3.94) | 0.014 | 2.52 (1.51, 4.20) | <0.001 | 2.44 (1.46, 4.08) | <0.001 |
| Overweight | 1.00 (0.79, 1.28) | 0.970 | 0.85 (0.67, 1.07) | 0.167 | 0.85 (0.66, 1.10) | 0.222 |
| Obesity | 0.90 (0.73, 1.12) | 0.358 | 0.83 (0.67, 1.03) | 0.091 | 0.85 (0.68, 1.07) | 0.165 |
| **Cancer-specific mortality (315/3,230)** | | | | | | |
| Variables | Model 1 |  | Model 2 |  | Model 3 | P value |
|  | HR (95% CI) | P value | HR (95% CI) | P value | HR (95% CI) |  |
| **ABSI** |  |  |  |  |  |  |
| Per 1-SD increase | 1.52 (1.33, 1.74) | <0.001 | 1.25 (1.07, 1.46) | 0.006 | 1.12 (0.96, 1.29) | 0.144 |
| Q1 | 1.00 (Reference) |  | 1.00 (Reference) |  | 1.00 (Reference) |  |
| Q2 | 2.59 (1.69, 3.98) | <0.001 | 2.08 (1.34, 3.24) | 0.001 | 1.85 (1.18, 2.88) | 0.007 |
| Q3 | 2.34 (1.43, 3.84) | <0.001 | 1.58 (0.94, 2.65) | 0.084 | 1.30 (0.77, 2.19) | 0.335 |
| Q4 | 3.71 (2.38, 5.78) | <0.001 | 2.18 (1.33, 3.56) | 0.002 | 1.64 (1.00, 2.69) | 0.052 |
| **BRI** |  |  |  |  |  |  |
| Per 1-unit increase | 1.02 (0.95, 1.10) | 0.577 | 1.01 (0.93, 1.09) | 0.888 | 0.99 (0.91, 1.07) | 0.766 |
| Q1 | 1.00 (Reference) |  | 1.00 (Reference) |  | 1.00 (Reference) |  |
| Q2 | 1.22 (0.83, 1.78) | 0.312 | 1.01 (0.69, 1.46) | 0.972 | 0.95 (0.67, 1.34) | 0.775 |
| Q3 | 1.19 (0.80, 1.75) | 0.392 | 0.91 (0.61, 1.34) | 0.631 | 0.84 (0.57, 1.24) | 0.387 |
| Q4 | 1.24 (0.81, 1.90) | 0.324 | 1.09 (0.72, 1.65) | 0.693 | 0.96 (0.64, 1.45) | 0.850 |
| **BMI** |  |  |  |  |  |  |
| Per 1 kg/m² increase | 0.99 (0.96, 1.02) | 0.442 | 0.99 (0.96, 1.02) | 0.536 | 0.99 (0.96, 1.03) | 0.635 |
| Normal weight | 1.00 (Reference) |  | 1.00 (Reference) |  | 1.00 (Reference) |  |
| Underweight | 2.50 (0.94, 6.66) | 0.066 | 2.84 (1.06, 7.65) | 0.039 | 2.80 (0.99, 7.93) | 0.053 |
| Overweight | 0.94 (0.60, 1.47) | 0.788 | 0.79 (0.51, 1.22) | 0.286 | 0.83 (0.52, 1.33) | 0.442 |
| Obesity | 0.92 (0.64, 1.34) | 0.678 | 0.82 (0.57, 1.20) | 0.311 | 0.86 (0.58, 1.26) | 0.435 |
| **CVD mortality (197/3,230)** | | | | | | |
| Variables | Model 1 |  | Model 2 |  | Model 3 | P value |
|  | HR (95% CI) | P value | HR (95% CI) | P value | HR (95% CI) |  |
| **ABSI** |  |  |  |  |  |  |
| Per 1-SD increase | 2.28 (1.90, 2.73) | <0.001 | 1.66 (1.33, 2.07) | <0.001 | 1.55 (1.26, 1.90) | <0.001 |
| Q1 | 1.00 (Reference) |  | 1.00 (Reference) |  | 1.00 (Reference) |  |
| Q2 | 2.05 (1.16, 3.64) | 0.014 | 1.42 (0.77, 2.62) | 0.266 | 1.32 (0.71, 2.48) | 0.382 |
| Q3 | 3.13 (1.72, 5.68) | <0.001 | 1.60 (0.80, 3.20) | 0.182 | 1.49 (0.75, 2.95) | 0.252 |
| Q4 | 7.27 (4.14, 12.78) | <0.001 | 2.85 (1.46, 5.58) | 0.002 | 2.45 (1.25, 4.78) | 0.009 |
| **BRI** |  |  |  |  |  |  |
| Per 1-unit increase | 1.12 (1.04, 1.20) | 0.002 | 1.11 (1.01, 1.22) | 0.032 | 1.09 (0.99, 1.20) | 0.094 |
| Q1 | 1.00 (Reference) |  | 1.00 (Reference) |  | 1.00 (Reference) |  |
| Q2 | 1.35 (0.82, 2.24) | 0.243 | 0.99 (0.62, 1.58) | 0.978 | 0.90 (0.57, 1.42) | 0.644 |
| Q3 | 2.46 (1.47, 4.13) | <0.001 | 1.60 (0.95, 2.68) | 0.076 | 1.56 (0.91, 2.65) | 0.103 |
| Q4 | 2.28 (1.32, 3.95) | 0.003 | 1.78 (1.01, 3.14) | 0.046 | 1.59 (0.89, 2.84) | 0.120 |
| **BMI** |  |  |  |  |  |  |
| Per 1 kg/m² increase | 1.00 (0.97, 1.04) | 0.929 | 1.01 (0.97, 1.05) | 0.623 | 1.01 (0.97, 1.05) | 0.652 |
| Normal weight | 1.00 (Reference) |  | 1.00 (Reference) |  | 1.00 (Reference) |  |
| Underweight | 1.57 (0.41, 6.02) | 0.509 | 1.95 (0.61, 6.21) | 0.257 | 1.94 (0.63, 5.97) | 0.250 |
| Overweight | 1.10 (0.66, 1.83) | 0.708 | 0.90 (0.55, 1.47) | 0.671 | 0.89 (0.54, 1.47) | 0.650 |
| Obesity | 1.17 (0.70, 1.96) | 0.545 | 1.06 (0.64, 1.74) | 0.820 | 1.11 (0.66, 1.87) | 0.694 |

*Data are presented as hazard ratios (HRs) with 95% confidence intervals (CIs).*

*Model 1: unadjusted model.*

*Model 2: adjusted for Age, Sex, Race/ethnicity.*

*Model 3: adjusted for* *Age, Sex, Race/ethnicity, Education level, PIR, Marital status, Alcohol status, Smoking status, cancer group, and years since first cancer diagnosis.*

*All analyses incorporated the NHANES complex survey design using WTMEC16YR, SDMVSTRA, and SDMVPSU. For cancer-specific and CVD mortality, deaths from other causes were censored.*

*Continuous* *estimates are reported per 1-SD increase in ABSI, per 1-unit increase in BRI, and per 1 kg/m² increase in BMI.*

**Table S4.** Survey-weighted Cox Model 4 sensitivity analysis (N=3,230).

| All-cause mortality (937/3,230) | | |
| --- | --- | --- |
| Variables | HR (95% CI) | P value |
| **ABSI** |  |  |
| Per 1-SD increase | 1.19 (1.07, 1.32) | 0.001 |
| Q1 | 1.00 (Reference) |  |
| Q2 | 1.18 (0.92, 1.51) | 0.200 |
| Q3 | 1.23 (0.89, 1.68) | 0.206 |
| Q4 | 1.42 (1.08, 1.87) | 0.012 |
| **BRI** |  |  |
| Per 1-unit increase | 0.97 (0.92, 1.01) | 0.160 |
| Q1 | 1.00 (Reference) |  |
| Q2 | 0.83 (0.68, 1.02) | 0.077 |
| Q3 | 0.85 (0.67, 1.07) | 0.165 |
| Q4 | 0.81 (0.65, 1.02) | 0.071 |
| **BMI** |  |  |
| Per 1 kg/m² increase | 0.98 (0.96, 0.99) | 0.006 |
| Normal weight | 1.00 (Reference) |  |
| Underweight | 2.46 (1.50, 4.03) | <0.001 |
| Overweight | 0.80 (0.62, 1.03) | 0.082 |
| Obesity | 0.71 (0.57, 0.90) | 0.004 |
| Cancer-specific mortality (315/3,230) | | |
| Variables | HR (95% CI) | P value |
| **ABSI** |  |  |
| Per 1-SD increase | 1.08 (0.93, 1.26) | 0.296 |
| Q1 | 1.00 (Reference) |  |
| Q2 | 1.78 (1.15, 2.74) | 0.009 |
| Q3 | 1.23 (0.73, 2.06) | 0.435 |
| Q4 | 1.51 (0.91, 2.49) | 0.108 |
| **BRI** |  |  |
| Per 1-unit increase | 0.96 (0.87, 1.05) | 0.343 |
| Q1 | 1.00 (Reference) |  |
| Q2 | 0.91 (0.64, 1.29) | 0.598 |
| Q3 | 0.74 (0.48, 1.13) | 0.162 |
| Q4 | 0.81 (0.52, 1.26) | 0.347 |
| **BMI** |  |  |
| Per 1 kg/m² increase | 0.98 (0.95, 1.02) | 0.329 |
| Normal weight | 1.00 (Reference) |  |
| Underweight | 2.80 (1.01, 7.77) | 0.049 |
| Overweight | 0.80 (0.51, 1.25) | 0.329 |
| Obesity | 0.76 (0.50, 1.13) | 0.176 |
| CVD mortality (197/3,230) | | |
| Variables | HR (95% CI) | P value |
| **ABSI** |  |  |
| Per 1-SD increase | 1.43 (1.16, 1.76) | <0.001 |
| Q1 | 1.00 (Reference) |  |
| Q2 | 1.13 (0.61, 2.10) | 0.688 |
| Q3 | 1.24 (0.63, 2.41) | 0.535 |
| Q4 | 1.94 (0.99, 3.79) | 0.052 |
| **BRI** |  |  |
| Per 1-unit increase | 1.04 (0.93, 1.16) | 0.533 |
| Q1 | 1.00 (Reference) |  |
| Q2 | 0.91 (0.58, 1.42) | 0.665 |
| Q3 | 1.25 (0.73, 2.14) | 0.421 |
| Q4 | 1.22 (0.69, 2.16) | 0.499 |
| **BMI** |  |  |
| Per 1 kg/m² increase | 0.99 (0.95, 1.04) | 0.732 |
| Normal weight | 1.00 (Reference) |  |
| Underweight | 1.90 (0.58, 6.18) | 0.287 |
| Overweight | 0.80 (0.49, 1.31) | 0.377 |
| Obesity | 0.85 (0.51, 1.43) | 0.546 |

*Data are presented as hazard ratios (HRs) with 95% confidence intervals (CIs).*

*Model 4 (extended cardiometabolic adjustment/sensitivity model): adjusted for Age, Sex, Race/ethnicity, Education level, PIR, Marital status, Alcohol status, Smoking status, cancer group, and years since first cancer diagnosis, hypertension, diabetes mellitus, and history of cardiovascular disease.*

*All analyses incorporated the NHANES complex survey design using WTMEC16YR, SDMVSTRA, and SDMVPSU. For cancer-specific and CVD mortality, deaths from other causes were censored.*

*Continuous estimates are reported per 1-SD increase in ABSI, per 1-unit increase in BRI, and per 1 kg/m² increase in BMI.*

**Table S5A.** Proportional-hazards diagnostics for Model 3.

| **Outcome** | **Indicator** | **Exposure PH diagnostic P** | **Global PH diagnostic P** | **Covariates flagged by PH diagnostics** |
| --- | --- | --- | --- | --- |
| All-cause mortality | ABSI | 0.760 | <0.001 | Age, Alcohol status, cancer group, years since first cancer diagnosis |
|  | BRI | 0.117 | <0.001 | Age, Alcohol status, cancer group, years since first cancer diagnosis |
|  | BMI | 0.184 | <0.001 | Age, Alcohol status, cancer group, years since first cancer diagnosis |
| Cancer-specific mortality | ABSI | 0.194 | 0.049 | cancer group |
|  | BRI | 0.726 | 0.114 | cancer group |
|  | BMI | 0.362 | 0.100 | cancer group |
| CVD mortality | ABSI | 0.607 | 0.036 | Age, Smoking status |
|  | BRI | 0.956 | 0.032 | Age, Smoking status |
|  | BMI | 0.761 | 0.029 | Age, Smoking status |

*Exposure-specific, model-term-specific, and global diagnostic P values were obtained by applying survival::cox.zph directly to each survey-weighted survey::svycoxph Model 3 fit (transform = "km" and terms = TRUE). No separate unweighted or conventional coxph model was fitted for this diagnostic. Although the underlying Cox models incorporated WTMEC16YR, SDMVSTRA, and SDMVPSU, cox.zph is not calibrated specifically for complex survey designs. These residual-based diagnostic P values were therefore interpreted as approximate and exploratory. Covariates flagged by the diagnostics informed the stratified sensitivity models presented in Table S5B.*

**Table S5B.** PH-stratified continuous-exposure sensitivity models.

| **Outcome** | **Index** | **Stratified variables** | **HR (95% CI)** | **P value** | **Exposure PH diagnostic P** | **Global PH diagnostic P** |
| --- | --- | --- | --- | --- | --- | --- |
| All-cause mortality | ABSI | Age, Alcohol status, cancer group, years since first cancer diagnosis (<5, 5–<10, ≥10 years) | 1.22 (1.09, 1.36) | <0.001 | 0.795 | 0.264 |
|  | BRI | Age, Alcohol status, cancer group, years since first cancer diagnosis (<5, 5–<10, ≥10 years) | 1.00 (0.96, 1.04) | 0.896 | 0.131 | 0.189 |
|  | BMI | Age, Alcohol status, cancer group, years since first cancer diagnosis (<5, 5–<10, ≥10 years) | 0.99 (0.97, 1.00) | 0.108 | 0.199 | 0.181 |
| Cancer-specific mortality | ABSI | cancer group | 1.12 (0.97, 1.30) | 0.132 | 0.147 | 0.419 |
|  | BRI | cancer group | 0.99 (0.91, 1.08) | 0.845 | 0.774 | 0.728 |
|  | BMI | cancer group | 0.99 (0.96, 1.03) | 0.698 | 0.385 | 0.642 |
| CVD mortality | ABSI | Age, Smoking status | 1.55 (1.25, 1.91) | <0.001 | 0.630 | 0.340 |
|  | BRI | Age, Smoking status | 1.08 (0.98, 1.18) | 0.105 | 0.827 | 0.262 |
|  | BMI | Age, Smoking status | 1.01 (0.97, 1.05) | 0.717 | 0.576 | 0.250 |

*Models incorporated the NHANES complex survey design and allowed separate baseline hazards across strata defined by the listed covariates. In the all-cause mortality models, years since first cancer diagnosis was stratified as <5, 5–<10, and ≥10 years, replacing its continuous adjustment term. The remaining Model 3 covariates were retained. Exposure-specific and global diagnostic P values for the stratified models were obtained by applying survival::cox.zph directly to the corresponding survey::svycoxph fits. Because cox.zph is not a survey-design-calibrated diagnostic procedure, these P values were interpreted as approximate and exploratory. The stratified models were treated as supporting sensitivity analyses rather than as proof that the proportional-hazards assumption holds.*

**Table S6.** Survey-weighted 12- and 24-month landmark sensitivity analyses.

| **Analysis** | **Outcome** | **N** | **Events** | **Index** | **Exposure level** | **HR (95% CI)** | **P value** |
| --- | --- | --- | --- | --- | --- | --- | --- |
| Primary | All-cause mortality | 3230 | 937 | ABSI | Per 1-SD increase | 1.24 (1.11, 1.38) | <0.001 |
| Primary | All-cause mortality | 3230 | 937 | ABSI | Q1 | 1.00 (Reference) |  |
| Primary | All-cause mortality | 3230 | 937 | ABSI | Q2 | 1.26 (0.98, 1.63) | 0.074 |
| Primary | All-cause mortality | 3230 | 937 | ABSI | Q3 | 1.32 (0.97, 1.81) | 0.080 |
| Primary | All-cause mortality | 3230 | 937 | ABSI | Q4 | 1.59 (1.21, 2.10) | <0.001 |
| Primary | All-cause mortality | 3230 | 937 | BRI | Per 1-unit increase | 1.01 (0.96, 1.05) | 0.739 |
| Primary | All-cause mortality | 3230 | 937 | BRI | Q1 | 1.00 (Reference) |  |
| Primary | All-cause mortality | 3230 | 937 | BRI | Q2 | 0.87 (0.71, 1.07) | 0.183 |
| Primary | All-cause mortality | 3230 | 937 | BRI | Q3 | 1.00 (0.80, 1.25) | 0.997 |
| Primary | All-cause mortality | 3230 | 937 | BRI | Q4 | 1.01 (0.81, 1.26) | 0.943 |
| Primary | All-cause mortality | 3230 | 937 | BMI | Per 1 kg/m^2^ increase | 0.99 (0.97, 1.01) | 0.208 |
| Primary | All-cause mortality | 3230 | 937 | BMI | Normal weight | 1.00 (Reference) |  |
| Primary | All-cause mortality | 3230 | 937 | BMI | Underweight | 2.44 (1.46, 4.08) | <0.001 |
| Primary | All-cause mortality | 3230 | 937 | BMI | Overweight | 0.85 (0.66, 1.10) | 0.222 |
| Primary | All-cause mortality | 3230 | 937 | BMI | Obesity | 0.85 (0.68, 1.07) | 0.165 |
| Primary | Cancer-specific mortality | 3230 | 315 | ABSI | Per 1-SD increase | 1.12 (0.96, 1.29) | 0.144 |
| Primary | Cancer-specific mortality | 3230 | 315 | ABSI | Q1 | 1.00 (Reference) |  |
| Primary | Cancer-specific mortality | 3230 | 315 | ABSI | Q2 | 1.85 (1.18, 2.88) | 0.007 |
| Primary | Cancer-specific mortality | 3230 | 315 | ABSI | Q3 | 1.30 (0.77, 2.19) | 0.335 |
| Primary | Cancer-specific mortality | 3230 | 315 | ABSI | Q4 | 1.64 (1.00, 2.69) | 0.052 |
| Primary | Cancer-specific mortality | 3230 | 315 | BRI | Per 1-unit increase | 0.99 (0.91, 1.07) | 0.766 |
| Primary | Cancer-specific mortality | 3230 | 315 | BRI | Q1 | 1.00 (Reference) |  |
| Primary | Cancer-specific mortality | 3230 | 315 | BRI | Q2 | 0.95 (0.67, 1.34) | 0.775 |
| Primary | Cancer-specific mortality | 3230 | 315 | BRI | Q3 | 0.84 (0.57, 1.24) | 0.387 |
| Primary | Cancer-specific mortality | 3230 | 315 | BRI | Q4 | 0.96 (0.64, 1.45) | 0.850 |
| Primary | Cancer-specific mortality | 3230 | 315 | BMI | Per 1 kg/m^2^ increase | 0.99 (0.96, 1.03) | 0.635 |
| Primary | Cancer-specific mortality | 3230 | 315 | BMI | Normal weight | 1.00 (Reference) |  |
| Primary | Cancer-specific mortality | 3230 | 315 | BMI | Underweight | 2.80 (0.99, 7.93) | 0.053 |
| Primary | Cancer-specific mortality | 3230 | 315 | BMI | Overweight | 0.83 (0.52, 1.33) | 0.442 |
| Primary | Cancer-specific mortality | 3230 | 315 | BMI | Obesity | 0.86 (0.58, 1.26) | 0.435 |
| Primary | CVD mortality | 3230 | 197 | ABSI | Per 1-SD increase | 1.55 (1.26, 1.90) | <0.001 |
| Primary | CVD mortality | 3230 | 197 | ABSI | Q1 | 1.00 (Reference) |  |
| Primary | CVD mortality | 3230 | 197 | ABSI | Q2 | 1.32 (0.71, 2.48) | 0.382 |
| Primary | CVD mortality | 3230 | 197 | ABSI | Q3 | 1.49 (0.75, 2.95) | 0.252 |
| Primary | CVD mortality | 3230 | 197 | ABSI | Q4 | 2.45 (1.25, 4.78) | 0.009 |
| Primary | CVD mortality | 3230 | 197 | BRI | Per 1-unit increase | 1.09 (0.99, 1.20) | 0.094 |
| Primary | CVD mortality | 3230 | 197 | BRI | Q1 | 1.00 (Reference) |  |
| Primary | CVD mortality | 3230 | 197 | BRI | Q2 | 0.90 (0.57, 1.42) | 0.644 |
| Primary | CVD mortality | 3230 | 197 | BRI | Q3 | 1.56 (0.91, 2.65) | 0.103 |
| Primary | CVD mortality | 3230 | 197 | BRI | Q4 | 1.59 (0.89, 2.84) | 0.120 |
| Primary | CVD mortality | 3230 | 197 | BMI | Per 1 kg/m^2^ increase | 1.01 (0.97, 1.05) | 0.652 |
| Primary | CVD mortality | 3230 | 197 | BMI | Normal weight | 1.00 (Reference) |  |
| Primary | CVD mortality | 3230 | 197 | BMI | Underweight | 1.94 (0.63, 5.97) | 0.250 |
| Primary | CVD mortality | 3230 | 197 | BMI | Overweight | 0.89 (0.54, 1.47) | 0.650 |
| Primary | CVD mortality | 3230 | 197 | BMI | Obesity | 1.11 (0.66, 1.87) | 0.694 |
| 12-month landmark | All-cause mortality | 3137 | 854 | ABSI | Per 1-SD increase | 1.25 (1.11, 1.39) | <0.001 |
| 12-month landmark | All-cause mortality | 3137 | 854 | ABSI | Q1 | 1.00 (Reference) |  |
| 12-month landmark | All-cause mortality | 3137 | 854 | ABSI | Q2 | 1.27 (0.99, 1.64) | 0.064 |
| 12-month landmark | All-cause mortality | 3137 | 854 | ABSI | Q3 | 1.34 (0.98, 1.83) | 0.067 |
| 12-month landmark | All-cause mortality | 3137 | 854 | ABSI | Q4 | 1.62 (1.22, 2.15) | <0.001 |
| 12-month landmark | All-cause mortality | 3137 | 854 | BRI | Per 1-unit increase | 1.01 (0.96, 1.06) | 0.699 |
| 12-month landmark | All-cause mortality | 3137 | 854 | BRI | Q1 | 1.00 (Reference) |  |
| 12-month landmark | All-cause mortality | 3137 | 854 | BRI | Q2 | 0.87 (0.71, 1.07) | 0.180 |
| 12-month landmark | All-cause mortality | 3137 | 854 | BRI | Q3 | 1.01 (0.80, 1.27) | 0.961 |
| 12-month landmark | All-cause mortality | 3137 | 854 | BRI | Q4 | 1.04 (0.81, 1.32) | 0.777 |
| 12-month landmark | All-cause mortality | 3137 | 854 | BMI | Per 1 kg/m^2^ increase | 0.99 (0.97, 1.01) | 0.236 |
| 12-month landmark | All-cause mortality | 3137 | 854 | BMI | Normal weight | 1.00 (Reference) |  |
| 12-month landmark | All-cause mortality | 3137 | 854 | BMI | Underweight | 2.54 (1.48, 4.38) | <0.001 |
| 12-month landmark | All-cause mortality | 3137 | 854 | BMI | Overweight | 0.88 (0.68, 1.14) | 0.326 |
| 12-month landmark | All-cause mortality | 3137 | 854 | BMI | Obesity | 0.87 (0.68, 1.11) | 0.259 |
| 12-month landmark | Cancer-specific mortality | 3137 | 270 | ABSI | Per 1-SD increase | 1.10 (0.94, 1.29) | 0.229 |
| 12-month landmark | Cancer-specific mortality | 3137 | 270 | ABSI | Q1 | 1.00 (Reference) |  |
| 12-month landmark | Cancer-specific mortality | 3137 | 270 | ABSI | Q2 | 1.82 (1.14, 2.91) | 0.012 |
| 12-month landmark | Cancer-specific mortality | 3137 | 270 | ABSI | Q3 | 1.26 (0.72, 2.20) | 0.424 |
| 12-month landmark | Cancer-specific mortality | 3137 | 270 | ABSI | Q4 | 1.64 (0.97, 2.76) | 0.065 |
| 12-month landmark | Cancer-specific mortality | 3137 | 270 | BRI | Per 1-unit increase | 0.97 (0.89, 1.06) | 0.519 |
| 12-month landmark | Cancer-specific mortality | 3137 | 270 | BRI | Q1 | 1.00 (Reference) |  |
| 12-month landmark | Cancer-specific mortality | 3137 | 270 | BRI | Q2 | 0.95 (0.66, 1.37) | 0.781 |
| 12-month landmark | Cancer-specific mortality | 3137 | 270 | BRI | Q3 | 0.91 (0.60, 1.37) | 0.643 |
| 12-month landmark | Cancer-specific mortality | 3137 | 270 | BRI | Q4 | 0.95 (0.60, 1.49) | 0.810 |
| 12-month landmark | Cancer-specific mortality | 3137 | 270 | BMI | Per 1 kg/m^2^ increase | 0.99 (0.95, 1.02) | 0.424 |
| 12-month landmark | Cancer-specific mortality | 3137 | 270 | BMI | Normal weight | 1.00 (Reference) |  |
| 12-month landmark | Cancer-specific mortality | 3137 | 270 | BMI | Underweight | 3.57 (1.27, 10.03) | 0.016 |
| 12-month landmark | Cancer-specific mortality | 3137 | 270 | BMI | Overweight | 0.94 (0.59, 1.51) | 0.795 |
| 12-month landmark | Cancer-specific mortality | 3137 | 270 | BMI | Obesity | 0.94 (0.61, 1.44) | 0.768 |
| 12-month landmark | CVD mortality | 3137 | 183 | ABSI | Per 1-SD increase | 1.56 (1.26, 1.92) | <0.001 |
| 12-month landmark | CVD mortality | 3137 | 183 | ABSI | Q1 | 1.00 (Reference) |  |
| 12-month landmark | CVD mortality | 3137 | 183 | ABSI | Q2 | 1.32 (0.70, 2.47) | 0.394 |
| 12-month landmark | CVD mortality | 3137 | 183 | ABSI | Q3 | 1.51 (0.76, 2.98) | 0.239 |
| 12-month landmark | CVD mortality | 3137 | 183 | ABSI | Q4 | 2.46 (1.28, 4.74) | 0.007 |
| 12-month landmark | CVD mortality | 3137 | 183 | BRI | Per 1-unit increase | 1.09 (0.99, 1.21) | 0.081 |
| 12-month landmark | CVD mortality | 3137 | 183 | BRI | Q1 | 1.00 (Reference) |  |
| 12-month landmark | CVD mortality | 3137 | 183 | BRI | Q2 | 0.94 (0.59, 1.50) | 0.805 |
| 12-month landmark | CVD mortality | 3137 | 183 | BRI | Q3 | 1.65 (0.96, 2.83) | 0.071 |
| 12-month landmark | CVD mortality | 3137 | 183 | BRI | Q4 | 1.68 (0.93, 3.04) | 0.085 |
| 12-month landmark | CVD mortality | 3137 | 183 | BMI | Per 1 kg/m^2^ increase | 1.01 (0.97, 1.06) | 0.620 |
| 12-month landmark | CVD mortality | 3137 | 183 | BMI | Normal weight | 1.00 (Reference) |  |
| 12-month landmark | CVD mortality | 3137 | 183 | BMI | Underweight | 1.86 (0.54, 6.38) | 0.321 |
| 12-month landmark | CVD mortality | 3137 | 183 | BMI | Overweight | 0.88 (0.52, 1.49) | 0.634 |
| 12-month landmark | CVD mortality | 3137 | 183 | BMI | Obesity | 1.12 (0.67, 1.88) | 0.664 |
| 24-month landmark | All-cause mortality | 2841 | 757 | ABSI | Per 1-SD increase | 1.25 (1.11, 1.41) | <0.001 |
| 24-month landmark | All-cause mortality | 2841 | 757 | ABSI | Q1 | 1.00 (Reference) |  |
| 24-month landmark | All-cause mortality | 2841 | 757 | ABSI | Q2 | 1.21 (0.93, 1.58) | 0.161 |
| 24-month landmark | All-cause mortality | 2841 | 757 | ABSI | Q3 | 1.39 (1.01, 1.93) | 0.046 |
| 24-month landmark | All-cause mortality | 2841 | 757 | ABSI | Q4 | 1.59 (1.19, 2.13) | 0.002 |
| 24-month landmark | All-cause mortality | 2841 | 757 | BRI | Per 1-unit increase | 1.02 (0.97, 1.08) | 0.360 |
| 24-month landmark | All-cause mortality | 2841 | 757 | BRI | Q1 | 1.00 (Reference) |  |
| 24-month landmark | All-cause mortality | 2841 | 757 | BRI | Q2 | 0.84 (0.68, 1.04) | 0.115 |
| 24-month landmark | All-cause mortality | 2841 | 757 | BRI | Q3 | 1.06 (0.83, 1.37) | 0.624 |
| 24-month landmark | All-cause mortality | 2841 | 757 | BRI | Q4 | 1.07 (0.82, 1.40) | 0.604 |
| 24-month landmark | All-cause mortality | 2841 | 757 | BMI | Per 1 kg/m^2^ increase | 0.99 (0.97, 1.01) | 0.587 |
| 24-month landmark | All-cause mortality | 2841 | 757 | BMI | Normal weight | 1.00 (Reference) |  |
| 24-month landmark | All-cause mortality | 2841 | 757 | BMI | Underweight | 2.75 (1.52, 4.98) | <0.001 |
| 24-month landmark | All-cause mortality | 2841 | 757 | BMI | Overweight | 0.85 (0.64, 1.14) | 0.279 |
| 24-month landmark | All-cause mortality | 2841 | 757 | BMI | Obesity | 0.91 (0.70, 1.18) | 0.478 |
| 24-month landmark | Cancer-specific mortality | 2841 | 221 | ABSI | Per 1-SD increase | 1.09 (0.91, 1.31) | 0.326 |
| 24-month landmark | Cancer-specific mortality | 2841 | 221 | ABSI | Q1 | 1.00 (Reference) |  |
| 24-month landmark | Cancer-specific mortality | 2841 | 221 | ABSI | Q2 | 1.81 (1.04, 3.15) | 0.034 |
| 24-month landmark | Cancer-specific mortality | 2841 | 221 | ABSI | Q3 | 1.35 (0.72, 2.53) | 0.351 |
| 24-month landmark | Cancer-specific mortality | 2841 | 221 | ABSI | Q4 | 1.65 (0.92, 2.97) | 0.093 |
| 24-month landmark | Cancer-specific mortality | 2841 | 221 | BRI | Per 1-unit increase | 0.98 (0.88, 1.08) | 0.639 |
| 24-month landmark | Cancer-specific mortality | 2841 | 221 | BRI | Q1 | 1.00 (Reference) |  |
| 24-month landmark | Cancer-specific mortality | 2841 | 221 | BRI | Q2 | 0.79 (0.53, 1.19) | 0.257 |
| 24-month landmark | Cancer-specific mortality | 2841 | 221 | BRI | Q3 | 0.91 (0.59, 1.39) | 0.655 |
| 24-month landmark | Cancer-specific mortality | 2841 | 221 | BRI | Q4 | 0.84 (0.50, 1.40) | 0.502 |
| 24-month landmark | Cancer-specific mortality | 2841 | 221 | BMI | Per 1 kg/m^2^ increase | 0.99 (0.95, 1.03) | 0.599 |
| 24-month landmark | Cancer-specific mortality | 2841 | 221 | BMI | Normal weight | 1.00 (Reference) |  |
| 24-month landmark | Cancer-specific mortality | 2841 | 221 | BMI | Underweight | 4.26 (1.44, 12.62) | 0.009 |
| 24-month landmark | Cancer-specific mortality | 2841 | 221 | BMI | Overweight | 0.86 (0.50, 1.47) | 0.581 |
| 24-month landmark | Cancer-specific mortality | 2841 | 221 | BMI | Obesity | 0.95 (0.59, 1.53) | 0.827 |
| 24-month landmark | CVD mortality | 2841 | 166 | ABSI | Per 1-SD increase | 1.56 (1.24, 1.96) | <0.001 |
| 24-month landmark | CVD mortality | 2841 | 166 | ABSI | Q1 | 1.00 (Reference) |  |
| 24-month landmark | CVD mortality | 2841 | 166 | ABSI | Q2 | 1.24 (0.62, 2.45) | 0.543 |
| 24-month landmark | CVD mortality | 2841 | 166 | ABSI | Q3 | 1.52 (0.74, 3.10) | 0.255 |
| 24-month landmark | CVD mortality | 2841 | 166 | ABSI | Q4 | 2.30 (1.13, 4.70) | 0.022 |
| 24-month landmark | CVD mortality | 2841 | 166 | BRI | Per 1-unit increase | 1.12 (1.01, 1.25) | 0.031 |
| 24-month landmark | CVD mortality | 2841 | 166 | BRI | Q1 | 1.00 (Reference) |  |
| 24-month landmark | CVD mortality | 2841 | 166 | BRI | Q2 | 1.03 (0.62, 1.70) | 0.913 |
| 24-month landmark | CVD mortality | 2841 | 166 | BRI | Q3 | 1.84 (1.01, 3.35) | 0.047 |
| 24-month landmark | CVD mortality | 2841 | 166 | BRI | Q4 | 2.06 (1.10, 3.88) | 0.025 |
| 24-month landmark | CVD mortality | 2841 | 166 | BMI | Per 1 kg/m^2^ increase | 1.02 (0.98, 1.07) | 0.353 |
| 24-month landmark | CVD mortality | 2841 | 166 | BMI | Normal weight | 1.00 (Reference) |  |
| 24-month landmark | CVD mortality | 2841 | 166 | BMI | Underweight | 2.57 (0.72, 9.15) | 0.146 |
| 24-month landmark | CVD mortality | 2841 | 166 | BMI | Overweight | 1.00 (0.55, 1.82) | 0.992 |
| 24-month landmark | CVD mortality | 2841 | 166 | BMI | Obesity | 1.34 (0.75, 2.39) | 0.322 |

*Participants were included only if they remained alive and under observation beyond the specified landmark. Participants who died or were censored at or before 12 or 24 months were excluded. Follow-up time was reset to zero at the corresponding landmark and accrued until death or administrative censoring through December 31, 2019. Sample sizes and event counts are unweighted. Models used the Model 3 covariates and incorporated WTMEC16YR, SDMVSTRA, and SDMVPSU.*

**Table S7.** Approximate survey-weighted Fine–Gray competing-risk sensitivity analyses for cancer-specific and CVD mortality (Model 3).

| **Outcome** | **Events** | **Competing events** | **Index** | **Exposure level** | **sHR (95% CI)** | **P value** |
| --- | --- | --- | --- | --- | --- | --- |
| Cancer-specific mortality | 315 | 622 | ABSI | Per 1-SD increase | 1.08 (0.93, 1.24) | 0.308 |
| Cancer-specific mortality | 315 | 622 | ABSI | Q1 | 1.00 (Reference) |  |
| Cancer-specific mortality | 315 | 622 | ABSI | Q2 | 1.90 (1.21, 2.98) | 0.005 |
| Cancer-specific mortality | 315 | 622 | ABSI | Q3 | 1.34 (0.79, 2.28) | 0.276 |
| Cancer-specific mortality | 315 | 622 | ABSI | Q4 | 1.56 (0.94, 2.59) | 0.083 |
| Cancer-specific mortality | 315 | 622 | BRI | Per 1-unit increase | 0.99 (0.91, 1.08) | 0.850 |
| Cancer-specific mortality | 315 | 622 | BRI | Q1 | 1.00 (Reference) |  |
| Cancer-specific mortality | 315 | 622 | BRI | Q2 | 1.02 (0.71, 1.47) | 0.909 |
| Cancer-specific mortality | 315 | 622 | BRI | Q3 | 0.87 (0.58, 1.31) | 0.515 |
| Cancer-specific mortality | 315 | 622 | BRI | Q4 | 0.99 (0.65, 1.52) | 0.973 |
| Cancer-specific mortality | 315 | 622 | BMI | Per 1 kg/m^2^ increase | 1.00 (0.97, 1.03) | 0.842 |
| Cancer-specific mortality | 315 | 622 | BMI | Normal weight | 1.00 (Reference) |  |
| Cancer-specific mortality | 315 | 622 | BMI | Underweight | 2.15 (0.74, 6.27) | 0.160 |
| Cancer-specific mortality | 315 | 622 | BMI | Overweight | 0.86 (0.54, 1.37) | 0.532 |
| Cancer-specific mortality | 315 | 622 | BMI | Obesity | 0.92 (0.62, 1.35) | 0.653 |
| CVD mortality | 197 | 740 | ABSI | Per 1-SD increase | 1.46 (1.21, 1.76) | <0.001 |
| CVD mortality | 197 | 740 | ABSI | Q1 | 1.00 (Reference) |  |
| CVD mortality | 197 | 740 | ABSI | Q2 | 1.30 (0.71, 2.39) | 0.395 |
| CVD mortality | 197 | 740 | ABSI | Q3 | 1.46 (0.75, 2.84) | 0.271 |
| CVD mortality | 197 | 740 | ABSI | Q4 | 2.31 (1.20, 4.43) | 0.012 |
| CVD mortality | 197 | 740 | BRI | Per 1-unit increase | 1.09 (0.99, 1.19) | 0.069 |
| CVD mortality | 197 | 740 | BRI | Q1 | 1.00 (Reference) |  |
| CVD mortality | 197 | 740 | BRI | Q2 | 0.98 (0.61, 1.56) | 0.918 |
| CVD mortality | 197 | 740 | BRI | Q3 | 1.67 (0.98, 2.84) | 0.059 |
| CVD mortality | 197 | 740 | BRI | Q4 | 1.66 (0.94, 2.93) | 0.081 |
| CVD mortality | 197 | 740 | BMI | Per 1 kg/m^2^ increase | 1.01 (0.98, 1.05) | 0.488 |
| CVD mortality | 197 | 740 | BMI | Normal weight | 1.00 (Reference) |  |
| CVD mortality | 197 | 740 | BMI | Underweight | 1.28 (0.39, 4.24) | 0.688 |
| CVD mortality | 197 | 740 | BMI | Overweight | 0.94 (0.57, 1.54) | 0.799 |
| CVD mortality | 197 | 740 | BMI | Obesity | 1.19 (0.72, 1.98) | 0.492 |

*Risk sets were expanded using survival::finegray, which generated the Fine–Gray weights (fgwt). The final analysis weight was calculated as WTMEC16YR × fgwt. The expanded data were analyzed using survey::svycoxph within a survey design specifying SDMVSTRA as the sampling strata and SDMVPSU as the primary sampling units, and variances were estimated using design-based linearization. Estimates are presented as subdistribution hazard ratios (sHRs) with 95% confidence intervals. For cancer-specific mortality, non-cancer deaths were treated as competing events; for CVD mortality, non-CVD deaths were treated as competing events. Because this combined implementation is not a dedicated Fine–Gray procedure developed specifically for complex survey data, the results should be interpreted as an approximate survey-weighted sensitivity analysis.*

**Table S8.** Survey-weighted restricted cubic spline knots and tests.

| **Outcome** | **Index** | **5th knot** | **35th knot** | **65th knot** | **95th knot** | **Reference median** | **P overall** | **P nonlinear** |
| --- | --- | --- | --- | --- | --- | --- | --- | --- |
| All-cause mortality | ABSI | 0.07536 | 0.08198 | 0.08574 | 0.09135 | 0.08384 | 0.001 | 0.385 |
|  | BRI | 2.66 | 4.64 | 6.18 | 9.92 | 5.40 | 0.542 | 0.348 |
|  | BMI | 20.20 | 25.80 | 30.28 | 40.83 | 27.90 | <0.001 | 0.002 |
| Cancer-specific mortality | ABSI | 0.07536 | 0.08198 | 0.08574 | 0.09135 | 0.08384 | 0.317 | 0.395 |
|  | BRI | 2.66 | 4.64 | 6.18 | 9.92 | 5.40 | 0.938 | 0.888 |
|  | BMI | 20.20 | 25.80 | 30.28 | 40.83 | 27.90 | 0.307 | 0.188 |
| CVD mortality | ABSI | 0.07536 | 0.08198 | 0.08574 | 0.09135 | 0.08384 | <0.001 | 0.626 |
|  | BRI | 2.66 | 4.64 | 6.18 | 9.92 | 5.40 | 0.147 | 0.347 |
|  | BMI | 20.20 | 25.80 | 30.28 | 40.83 | 27.90 | 0.291 | 0.161 |

**Table S9A.** Exploratory ABSI subgroup interaction tests.

| **Outcome** | **Subgroup** | **P for interaction** | **FDR q within outcome (12 tests)** | **FDR q across all tests (36 tests)** |
| --- | --- | --- | --- | --- |
| All-cause mortality | Age | 0.060 | 0.180 | 0.240 |
| All-cause mortality | Sex | 0.396 | 0.528 | 0.619 |
| All-cause mortality | Race/ethnicity | 0.459 | 0.551 | 0.662 |
| All-cause mortality | Education level | 0.246 | 0.369 | 0.402 |
| All-cause mortality | Poverty-income ratio | 0.235 | 0.369 | 0.402 |
| All-cause mortality | Marital status | 0.094 | 0.189 | 0.283 |
| All-cause mortality | Alcohol status | 0.601 | 0.656 | 0.710 |
| All-cause mortality | Smoking status | 0.690 | 0.690 | 0.749 |
| All-cause mortality | Hypertension | 0.060 | 0.180 | 0.240 |
| All-cause mortality | Diabetes mellitus | 0.041 | 0.180 | 0.240 |
| All-cause mortality | CVD history | 0.046 | 0.180 | 0.240 |
| All-cause mortality | Cancer group | 0.083 | 0.189 | 0.273 |
| Cancer-specific mortality | Age | 0.031 | 0.337 | 0.240 |
| Cancer-specific mortality | Sex | 0.571 | 0.758 | 0.710 |
| Cancer-specific mortality | Race/ethnicity | 0.803 | 0.876 | 0.826 |
| Cancer-specific mortality | Education level | 0.631 | 0.758 | 0.710 |
| Cancer-specific mortality | Poverty-income ratio | 0.516 | 0.758 | 0.701 |
| Cancer-specific mortality | Marital status | 0.237 | 0.569 | 0.402 |
| Cancer-specific mortality | Alcohol status | 0.438 | 0.758 | 0.657 |
| Cancer-specific mortality | Smoking status | 0.213 | 0.569 | 0.402 |
| Cancer-specific mortality | Hypertension | 0.056 | 0.337 | 0.240 |
| Cancer-specific mortality | Diabetes mellitus | 0.609 | 0.758 | 0.710 |
| Cancer-specific mortality | CVD history | 0.880 | 0.880 | 0.880 |
| Cancer-specific mortality | Cancer group | 0.193 | 0.569 | 0.402 |
| CVD mortality | Age | 0.526 | 0.631 | 0.701 |
| CVD mortality | Sex | 0.707 | 0.707 | 0.749 |
| CVD mortality | Race/ethnicity | 0.034 | 0.138 | 0.240 |
| CVD mortality | Education level | 0.074 | 0.223 | 0.268 |
| CVD mortality | Poverty-income ratio | 0.192 | 0.309 | 0.402 |
| CVD mortality | Marital status | 0.230 | 0.309 | 0.402 |
| CVD mortality | Alcohol status | 0.611 | 0.667 | 0.710 |
| CVD mortality | Smoking status | 0.170 | 0.309 | 0.402 |
| CVD mortality | Hypertension | 0.232 | 0.309 | 0.402 |
| CVD mortality | Diabetes mellitus | 0.003 | 0.019 | 0.056 |
| CVD mortality | CVD history | <0.001 | 0.001 | 0.004 |
| CVD mortality | Cancer group | 0.120 | 0.287 | 0.331 |

*Interaction P values were obtained from formal multiplicative interaction terms in survey-weighted Model 3. Benjamini-Hochberg false-discovery-rate (FDR) q values were calculated both within each outcome (12 subgroup factors) and across all 36 ABSI interaction tests (12 subgroup factors × three outcomes). Subgroup-specific estimates are presented in Table S9B; cancer-group-specific estimates are also summarized in Table S12A and Figure S5. All analyses were exploratory.*

**Table S9B.** Continuous ABSI estimates within exploratory subgroups.

| **Outcome** | **Subgroup** | **Level** | **N** | **Events** | **HR (95% CI)** | **P value** | **P interaction** | **FDR q** |
| --- | --- | --- | --- | --- | --- | --- | --- | --- |
| All-cause mortality | Age | <65 | 1320 | 159 | 1.37 (1.11, 1.70) | 0.004 | 0.060 | 0.240 |
| All-cause mortality | Age | ≥65 | 1910 | 778 | 1.21 (1.08, 1.36) | 0.001 | 0.060 | 0.240 |
| All-cause mortality | Sex | Male | 1530 | 553 | 1.39 (1.20, 1.61) | <0.001 | 0.396 | 0.619 |
| All-cause mortality | Sex | Female | 1700 | 384 | 1.17 (1.03, 1.33) | 0.015 | 0.396 | 0.619 |
| All-cause mortality | Race/ethnicity | Non-Hispanic White | 2255 | 731 | 1.24 (1.11, 1.39) | <0.001 | 0.459 | 0.662 |
| All-cause mortality | Race/ethnicity | Mexican American | 203 | 34 | 0.85 (0.42, 1.69) | 0.639 | 0.459 | 0.662 |
| All-cause mortality | Race/ethnicity | Non-Hispanic Black | 453 | 133 | 1.35 (1.07, 1.69) | 0.011 | 0.459 | 0.662 |
| All-cause mortality | Race/ethnicity | Other | 319 | 39 | 1.24 (0.70, 2.21) | 0.457 | 0.459 | 0.662 |
| All-cause mortality | Education level | >High school | 2555 | 651 | 1.26 (1.12, 1.40) | <0.001 | 0.246 | 0.402 |
| All-cause mortality | Education level | Completed high school | 391 | 156 | 1.22 (0.95, 1.55) | 0.113 | 0.246 | 0.402 |
| All-cause mortality | Education level | <High school | 284 | 130 | 1.13 (0.79, 1.62) | 0.511 | 0.246 | 0.402 |
| All-cause mortality | Poverty-income ratio | <1.30 | 765 | 258 | 1.37 (1.14, 1.65) | <0.001 | 0.235 | 0.402 |
| All-cause mortality | Poverty-income ratio | 1.30-3.49 | 1329 | 447 | 1.14 (0.96, 1.35) | 0.122 | 0.235 | 0.402 |
| All-cause mortality | Poverty-income ratio | ≥3.5 | 1136 | 232 | 1.38 (1.13, 1.68) | 0.002 | 0.235 | 0.402 |
| All-cause mortality | Marital status | Married/living with partner | 1968 | 509 | 1.33 (1.15, 1.53) | <0.001 | 0.094 | 0.283 |
| All-cause mortality | Marital status | Living alone | 1262 | 428 | 1.17 (1.00, 1.36) | 0.047 | 0.094 | 0.283 |
| All-cause mortality | Alcohol status | No | 1260 | 469 | 1.26 (1.10, 1.45) | <0.001 | 0.601 | 0.710 |
| All-cause mortality | Alcohol status | Yes | 1970 | 468 | 1.24 (1.08, 1.41) | 0.002 | 0.601 | 0.710 |
| All-cause mortality | Smoking status | Never | 1416 | 343 | 1.14 (0.97, 1.34) | 0.103 | 0.690 | 0.749 |
| All-cause mortality | Smoking status | Former | 1302 | 464 | 1.33 (1.17, 1.52) | <0.001 | 0.690 | 0.749 |
| All-cause mortality | Smoking status | Current | 512 | 130 | 1.26 (0.95, 1.66) | 0.113 | 0.690 | 0.749 |
| All-cause mortality | Hypertension | No | 1160 | 226 | 1.24 (1.03, 1.50) | 0.025 | 0.060 | 0.240 |
| All-cause mortality | Hypertension | Yes | 2070 | 711 | 1.20 (1.07, 1.35) | 0.002 | 0.060 | 0.240 |
| All-cause mortality | Diabetes mellitus | No | 2431 | 656 | 1.25 (1.12, 1.40) | <0.001 | 0.041 | 0.240 |
| All-cause mortality | Diabetes mellitus | Yes | 799 | 281 | 1.06 (0.85, 1.31) | 0.606 | 0.041 | 0.240 |
| All-cause mortality | CVD history | No | 2473 | 596 | 1.28 (1.14, 1.44) | <0.001 | 0.046 | 0.240 |
| All-cause mortality | CVD history | Yes | 757 | 341 | 1.08 (0.90, 1.31) | 0.406 | 0.046 | 0.240 |
| Cancer-specific mortality | Age | <65 | 1320 | 82 | 1.46 (1.11, 1.94) | 0.008 | 0.031 | 0.240 |
| Cancer-specific mortality | Age | ≥65 | 1910 | 233 | 0.99 (0.83, 1.18) | 0.902 | 0.031 | 0.240 |
| Cancer-specific mortality | Sex | Male | 1530 | 200 | 1.20 (0.97, 1.49) | 0.089 | 0.571 | 0.710 |
| Cancer-specific mortality | Sex | Female | 1700 | 115 | 1.04 (0.83, 1.31) | 0.710 | 0.571 | 0.710 |
| Cancer-specific mortality | Race/ethnicity | Non-Hispanic White | 2255 | 217 | 1.11 (0.93, 1.31) | 0.252 | 0.803 | 0.826 |
| Cancer-specific mortality | Race/ethnicity | Mexican American | 203 | 15*^*^* | Not estimable |  | 0.803 | 0.826 |
| Cancer-specific mortality | Race/ethnicity | Non-Hispanic Black | 453 | 61 | 1.36 (1.01, 1.81) | 0.040 | 0.803 | 0.826 |
| Cancer-specific mortality | Race/ethnicity | Other | 319 | 22 | 1.01 (0.57, 1.77) | 0.975 | 0.803 | 0.826 |
| Cancer-specific mortality | Education level | >High school | 2555 | 218 | 1.12 (0.95, 1.34) | 0.183 | 0.631 | 0.710 |
| Cancer-specific mortality | Education level | Completed high school | 391 | 57 | 1.20 (0.75, 1.90) | 0.448 | 0.631 | 0.710 |
| Cancer-specific mortality | Education level | <High school | 284 | 40 | 0.90 (0.54, 1.50) | 0.693 | 0.631 | 0.710 |
| Cancer-specific mortality | Poverty-income ratio | <1.30 | 765 | 95 | 1.40 (1.07, 1.83) | 0.015 | 0.516 | 0.701 |
| Cancer-specific mortality | Poverty-income ratio | 1.30-3.49 | 1329 | 139 | 1.03 (0.83, 1.29) | 0.778 | 0.516 | 0.701 |
| Cancer-specific mortality | Poverty-income ratio | ≥3.5 | 1136 | 81 | 0.96 (0.69, 1.33) | 0.794 | 0.516 | 0.701 |
| Cancer-specific mortality | Marital status | Married/living with partner | 1968 | 171 | 1.13 (0.91, 1.40) | 0.265 | 0.237 | 0.402 |
| Cancer-specific mortality | Marital status | Living alone | 1262 | 144 | 1.10 (0.91, 1.33) | 0.337 | 0.237 | 0.402 |
| Cancer-specific mortality | Alcohol status | No | 1260 | 149 | 1.08 (0.87, 1.34) | 0.503 | 0.438 | 0.657 |
| Cancer-specific mortality | Alcohol status | Yes | 1970 | 166 | 1.15 (0.93, 1.44) | 0.197 | 0.438 | 0.657 |
| Cancer-specific mortality | Smoking status | Never | 1416 | 102 | 0.90 (0.73, 1.09) | 0.281 | 0.213 | 0.402 |
| Cancer-specific mortality | Smoking status | Former | 1302 | 161 | 1.18 (0.98, 1.43) | 0.080 | 0.213 | 0.402 |
| Cancer-specific mortality | Smoking status | Current | 512 | 52 | 1.45 (0.93, 2.24) | 0.099 | 0.213 | 0.402 |
| Cancer-specific mortality | Hypertension | No | 1160 | 85 | 1.30 (1.01, 1.68) | 0.043 | 0.056 | 0.240 |
| Cancer-specific mortality | Hypertension | Yes | 2070 | 230 | 1.03 (0.85, 1.24) | 0.751 | 0.056 | 0.240 |
| Cancer-specific mortality | Diabetes mellitus | No | 2431 | 220 | 1.11 (0.93, 1.33) | 0.244 | 0.609 | 0.710 |
| Cancer-specific mortality | Diabetes mellitus | Yes | 799 | 95 | 1.01 (0.74, 1.37) | 0.971 | 0.609 | 0.710 |
| Cancer-specific mortality | CVD history | No | 2473 | 219 | 1.11 (0.92, 1.33) | 0.294 | 0.880 | 0.880 |
| Cancer-specific mortality | CVD history | Yes | 757 | 96 | 1.13 (0.82, 1.56) | 0.461 | 0.880 | 0.880 |
| CVD mortality | Age | <65 | 1320 | 19 | 1.52 (0.89, 2.59) | 0.121 | 0.526 | 0.701 |
| CVD mortality | Age | ≥65 | 1910 | 178 | 1.54 (1.23, 1.93) | <0.001 | 0.526 | 0.701 |
| CVD mortality | Sex | Male | 1530 | 125 | 1.66 (1.23, 2.24) | <0.001 | 0.707 | 0.749 |
| CVD mortality | Sex | Female | 1700 | 72 | 1.49 (1.14, 1.94) | 0.004 | 0.707 | 0.749 |
| CVD mortality | Race/ethnicity | Non-Hispanic White | 2255 | 170 | 1.57 (1.26, 1.95) | <0.001 | 0.034 | 0.240 |
| CVD mortality | Race/ethnicity | Mexican American | 203 | 5*^*^* | Not estimable |  | 0.034 | 0.240 |
| CVD mortality | Race/ethnicity | Non-Hispanic Black | 453 | 20 | 1.71 (1.11, 2.65) | 0.016 | 0.034 | 0.240 |
| CVD mortality | Race/ethnicity | Other | 319 | 2*^*^* | Not estimable |  | 0.034 | 0.240 |
| CVD mortality | Education level | >High school | 2555 | 144 | 1.65 (1.29, 2.11) | <0.001 | 0.074 | 0.268 |
| CVD mortality | Education level | Completed high school | 391 | 22 | 1.36 (0.87, 2.14) | 0.178 | 0.074 | 0.268 |
| CVD mortality | Education level | <High school | 284 | 31 | 1.01 (0.62, 1.65) | 0.956 | 0.074 | 0.268 |
| CVD mortality | Poverty-income ratio | <1.30 | 765 | 49 | 1.85 (1.18, 2.89) | 0.007 | 0.192 | 0.402 |
| CVD mortality | Poverty-income ratio | 1.30-3.49 | 1329 | 96 | 1.34 (0.98, 1.83) | 0.065 | 0.192 | 0.402 |
| CVD mortality | Poverty-income ratio | ≥3.5 | 1136 | 52 | 2.01 (1.33, 3.03) | <0.001 | 0.192 | 0.402 |
| CVD mortality | Marital status | Married/living with partner | 1968 | 108 | 1.71 (1.25, 2.35) | <0.001 | 0.230 | 0.402 |
| CVD mortality | Marital status | Living alone | 1262 | 89 | 1.38 (1.00, 1.90) | 0.050 | 0.230 | 0.402 |
| CVD mortality | Alcohol status | No | 1260 | 100 | 1.57 (1.27, 1.93) | <0.001 | 0.611 | 0.710 |
| CVD mortality | Alcohol status | Yes | 1970 | 97 | 1.52 (1.10, 2.11) | 0.012 | 0.611 | 0.710 |
| CVD mortality | Smoking status | Never | 1416 | 79 | 1.40 (0.97, 2.03) | 0.072 | 0.170 | 0.402 |
| CVD mortality | Smoking status | Former | 1302 | 96 | 1.43 (1.07, 1.92) | 0.016 | 0.170 | 0.402 |
| CVD mortality | Smoking status | Current | 512 | 22 | 2.04 (1.43, 2.91) | <0.001 | 0.170 | 0.402 |
| CVD mortality | Hypertension | No | 1160 | 36 | 1.84 (1.08, 3.14) | 0.024 | 0.232 | 0.402 |
| CVD mortality | Hypertension | Yes | 2070 | 161 | 1.44 (1.16, 1.80) | <0.001 | 0.232 | 0.402 |
| CVD mortality | Diabetes mellitus | No | 2431 | 132 | 1.87 (1.44, 2.42) | <0.001 | 0.003 | 0.056 |
| CVD mortality | Diabetes mellitus | Yes | 799 | 65 | 0.98 (0.65, 1.49) | 0.937 | 0.003 | 0.056 |
| CVD mortality | CVD history | No | 2473 | 89 | 2.39 (1.84, 3.12) | <0.001 | <0.001 | 0.004 |
| CVD mortality | CVD history | Yes | 757 | 108 | 0.98 (0.73, 1.33) | 0.914 | <0.001 | 0.004 |

*Sparse singular strata are reported as not estimable. Models were adjusted for Model 3 covariates except the stratifying variable. Interaction P values were obtained from formal multiplicative interaction terms. FDR q values were calculated across all 36 ABSI subgroup interaction tests (12 subgroup factors × three outcomes); Table S9A additionally presents FDR q values calculated within each outcome across 12 tests. Within-subgroup P values are unadjusted. Cancer-group-specific estimates are presented separately in Table S12A and Figure S5 and are not additional hypothesis tests. *Fewer than 20 deaths; interpret cautiously. All models incorporated WTMEC16YR, SDMVSTRA, and SDMVPSU. Counts are unweighted.*

**Table S10.** Variable-specific missingness among 4,260 cancer survivors before sequential exclusions.

| **Variable** | **Missing n** | **Missing %** |
| --- | --- | --- |
| Height | 281 | 6.60 |
| Weight | 269 | 6.31 |
| BMI | 288 | 6.76 |
| Waist circumference | 534 | 12.54 |
| BRI | 544 | 12.77 |
| ABSI | 545 | 12.79 |
| Mortality status | 3 | 0.07 |
| Follow-up months | 197 | 4.62 |
| Age | 0 | 0.00 |
| Sex | 0 | 0.00 |
| Race/ethnicity | 0 | 0.00 |
| Education level | 6 | 0.14 |
| PIR | 387 | 9.08 |
| Marital status | 6 | 0.14 |
| Alcohol status | 489 | 11.48 |
| Smoking status | 4 | 0.09 |
| Hypertension | 0 | 0.00 |
| diabetes mellitus | 0 | 0.00 |
| CVD history | 0 | 0.00 |
| years since first diagnosis | 71 | 1.67 |

*Missingness is variable-specific and therefore not additive. Sequential exclusions were 545 for anthropometric data, 1 for mortality follow-up after anthropometric restriction, 484 with missing covariate data, yielding N=3,230. The covariate-only multiple-imputation sensitivity analysis retained 3,714 participants with complete anthropometric measurements and eligible mortality follow-up.*

**Table S10A.** Number of cancer types reported by participants in the complete-case analytic sample.

| **Number of reported cancer types** | **n** | **Unweighted %** | **Survey-weighted %** |
| --- | --- | --- | --- |
| One | 2,894 | 89.6 | 89.7 |
| Two | 297 | 9.2 | 9.2 |
| Three | 39 | 1.2 | 1.2 |

*Cancer types were identified from the available NHANES cancer questionnaire responses (MCQ230A-MCQ230D). Overall, 336 participants reported at least two cancer types (unweighted, 10.4%; survey-weighted, 10.3%). For the mutually exclusive cancer-group variable used in the multivariable models, participants were classified according to the first cancer type recorded in MCQ230A; this was not necessarily the earliest diagnosed, most recent, or active cancer. Survey-weighted percentages were calculated using WTMEC16YR.*

**Table S11.** Comparison of survey-weighted Cox estimates between complete-case and covariate-only multiple-imputation analyses for continuous obesity indices.

| **Outcome** | **Model** | **Index (scale)** | **Complete-case HR (95% CI)** | **P value** | **Multiple-imputation HR (95% CI)** | **P value** |
| --- | --- | --- | --- | --- | --- | --- |
| All-cause mortality | Model 3 | ABSI (per 1-SD) | 1.24 (1.11–1.38) | <0.001 | 1.25 (1.14–1.38) | <0.001 |
|  |  | BRI (per 1 unit) | 1.01 (0.96–1.05) | 0.739 | 1.01 (0.97–1.05) | 0.637 |
|  |  | BMI (per 1 kg/m²) | 0.99 (0.97–1.01) | 0.208 | 0.99 (0.97–1.01) | 0.190 |
|  | Model 4 | ABSI (per 1-SD) | 1.19 (1.07–1.32) | 0.001 | 1.22 (1.10–1.34) | <0.001 |
|  |  | BRI (per 1 unit) | 0.97 (0.92–1.01) | 0.160 | 0.97 (0.93–1.02) | 0.252 |
|  |  | BMI (per 1 kg/m²) | 0.98 (0.96–0.99) | 0.006 | 0.98 (0.96–0.99) | 0.006 |
| Cancer-specific mortality | Model 3 | ABSI (per 1-SD) | 1.12 (0.96–1.29) | 0.144 | 1.11 (0.96–1.28) | 0.160 |
|  |  | BRI (per 1 unit) | 0.99 (0.91–1.07) | 0.766 | 0.99 (0.92–1.06) | 0.725 |
|  |  | BMI (per 1 kg/m²) | 0.99 (0.96–1.03) | 0.635 | 0.99 (0.97–1.02) | 0.638 |
|  | Model 4 | ABSI (per 1-SD) | 1.08 (0.93–1.26) | 0.296 | 1.09 (0.94–1.26) | 0.262 |
|  |  | BRI (per 1 unit) | 0.96 (0.87–1.05) | 0.343 | 0.96 (0.89–1.05) | 0.380 |
|  |  | BMI (per 1 kg/m²) | 0.98 (0.95–1.02) | 0.329 | 0.99 (0.96–1.02) | 0.379 |
| CVD mortality | Model 3 | ABSI (per 1-SD) | 1.55 (1.26–1.90) | <0.001 | 1.47 (1.22–1.78) | <0.001 |
|  |  | BRI (per 1 unit) | 1.09 (0.99–1.20) | 0.094 | 1.09 (1.00–1.19) | 0.062 |
|  |  | BMI (per 1 kg/m²) | 1.01 (0.97–1.05) | 0.652 | 1.01 (0.97–1.05) | 0.567 |
|  | Model 4 | ABSI (per 1-SD) | 1.43 (1.16–1.76) | <0.001 | 1.38 (1.15–1.67) | <0.001 |
|  |  | BRI (per 1 unit) | 1.04 (0.93–1.16) | 0.533 | 1.04 (0.94–1.15) | 0.422 |
|  |  | BMI (per 1 kg/m²) | 0.99 (0.95–1.04) | 0.732 | 0.99 (0.95–1.04) | 0.764 |

*Note: The complete-case analysis included 3,230 participants with 937 all-cause, 315 cancer-specific, and 197 CVD deaths; the multiple-imputation analysis included 3,714 participants with 1,079, 358, and 228 deaths, respectively. Model 3 adjusted for Age, Sex, Race/ethnicity, Education level, PIR, Marital status, Alcohol status, Smoking status, cancer group, and years since first cancer diagnosis. Model 4 additionally adjusted for hypertension, diabetes mellitus, and history of cardiovascular disease. All models incorporated WTMEC16YR, SDMVSTRA, and SDMVPSU. Multiple-imputation estimates were pooled using Rubin’s rules. All 18 comparisons showed the same direction of association and P<0.05 classification as the corresponding complete-case estimates. Convergence was assessed using trace plots of the chain means and standard deviations. No sustained drift or clear separation between imputation chains was observed. Abbreviations: ABSI, a body shape index; BRI, body roundness index; BMI, body mass index; CI, confidence interval; CVD, cardiovascular disease; HR, hazard ratio; PIR, poverty-income ratio.*

**Table S12A.** Exploratory cancer-group-specific associations of ABSI with mortality in survey-weighted Model 3.

| **Outcome** | **Cancer group** | **N** | **Events** | **HR (95% CI)** | **P value** | **P interaction** | **FDR q (36 tests)** |
| --- | --- | --- | --- | --- | --- | --- | --- |
| All-cause mortality | Breast | 495 | 133 | 1.31 (1.10, 1.56) | 0.002 | 0.083 | 0.273 |
| All-cause mortality | Prostate | 495 | 174 | 1.39 (1.06, 1.83) | 0.016 |  |  |
| All-cause mortality | Non-melanoma/unspecified skin | 738 | 207 | 1.36 (1.09, 1.69) | 0.006 |  |  |
| All-cause mortality | Colorectal | 205 | 84 | 0.83 (0.62, 1.12) | 0.218 |  |  |
| All-cause mortality | Gynecologic | 416 | 57 | 1.22 (0.88, 1.70) | 0.230 |  |  |
| All-cause mortality | Melanoma | 191 | 45 | 1.62 (1.06, 2.50) | 0.027 |  |  |
| All-cause mortality | Hematologic | 123 | 44 | 0.92 (0.57, 1.49) | 0.730 |  |  |
| All-cause mortality | Lung | 73 | 36 | 1.02 (0.61, 1.71) | 0.943 |  |  |
| All-cause mortality | Urinary | 126 | 46 | 1.17 (0.76, 1.81) | 0.475 |  |  |
| All-cause mortality | Other/unknown | 368 | 111 | 1.17 (0.94, 1.45) | 0.154 |  |  |
| Cancer-specific mortality | Breast | 495 | 48 | 1.17 (0.86, 1.60) | 0.321 | 0.193 | 0.402 |
| Cancer-specific mortality | Prostate | 495 | 64 | 1.29 (0.86, 1.94) | 0.215 |  |  |
| Cancer-specific mortality | Non-melanoma/unspecified skin | 738 | 49 | 1.38 (0.94, 2.02) | 0.100 |  |  |
| Cancer-specific mortality | Colorectal | 205 | 23 | 0.83 (0.51, 1.36) | 0.463 |  |  |
| Cancer-specific mortality | Gynecologic | 416 | 12^*^ | 1.79 (0.93, 3.47) | 0.082 |  |  |
| Cancer-specific mortality | Melanoma | 191 | 11^*^ | 1.45 (0.62, 3.37) | 0.387 |  |  |
| Cancer-specific mortality | Hematologic | 123 | 30 | 0.95 (0.64, 1.40) | 0.785 |  |  |
| Cancer-specific mortality | Lung | 73 | 20 | 0.88 (0.54, 1.44) | 0.620 |  |  |
| Cancer-specific mortality | Urinary | 126 | 16^*^ | 1.14 (0.60, 2.14) | 0.694 |  |  |
| Cancer-specific mortality | Other/unknown | 368 | 42 | 0.84 (0.60, 1.16) | 0.283 |  |  |
| CVD mortality | Breast | 495 | 21 | 1.51 (1.04, 2.20) | 0.032 | 0.120 | 0.331 |
| CVD mortality | Prostate | 495 | 43 | 1.35 (0.75, 2.43) | 0.314 |  |  |
| CVD mortality | Non-melanoma/unspecified skin | 738 | 46 | 1.68 (1.14, 2.49) | 0.009 |  |  |
| CVD mortality | Colorectal | 205 | 21 | 0.75 (0.40, 1.39) | 0.361 |  |  |
| CVD mortality | Gynecologic | 416 | 15^*^ | 2.12 (1.23, 3.66) | 0.007 |  |  |
| CVD mortality | Melanoma | 191 | 13^*^ | 2.29 (0.56, 9.34) | 0.247 |  |  |
| CVD mortality | Hematologic | 123 | 5^*^ | 4.69 (2.00, 11.00) | <0.001 |  |  |
| CVD mortality | Lung | 73 | 3^*^ | 2.93 (0.65, 13.13) | 0.160 |  |  |
| CVD mortality | Urinary | 126 | 9^*^ | 1.41 (0.44, 4.57) | 0.564 |  |  |
| CVD mortality | Other/unknown | 368 | 21 | 1.64 (1.07, 2.52) | 0.023 |  |  |

*HRs were derived as linear contrasts from a pooled Cox model containing the continuous obesity indicator × cancer-group interaction, with common coefficients for the other covariates. They are not estimates from independently fitted fully adjusted models in each cancer group. Model 3 adjusted for Age (categorical), Sex, Race/ethnicity, Education level, PIR (categorical), Marital status, Alcohol status, Smoking status, Cancer group, and Years since first cancer diagnosis (continuous).*

*ABSI estimates are reported per 1-SD increase, using the same standardization as in the main analysis. All models incorporated WTMEC16YR, SDMVSTRA, and SDMVPSU. N and death counts are unweighted.*

** Fewer than 20 deaths: estimates require particular caution; this is a descriptive flag, not a universal validity threshold. All within-group P values are descriptive and unadjusted for multiple testing. Global interaction P values assess heterogeneity across all ten cancer groups; see Table S13 for multiplicity families.*

*Abbreviations: ABSI, a body shape index; BRI, body roundness index; BMI, body mass index; CI, confidence interval; CVD, cardiovascular disease; HR, hazard ratio; BH, Benjamini–Hochberg.*

**Table S12B.** Exploratory cancer-group-specific associations of BRI with mortality in survey-weighted Model 3.

| **Outcome** | **Cancer group** | **N** | **Events** | **HR (95% CI)** | **P value** | **P interaction** | **FDR q (9 tests)** |
| --- | --- | --- | --- | --- | --- | --- | --- |
| All-cause mortality | Breast | 495 | 133 | 1.01 (0.93, 1.10) | 0.836 | 0.273 | 0.351 |
| All-cause mortality | Prostate | 495 | 174 | 1.08 (0.93, 1.25) | 0.292 |  |  |
| All-cause mortality | Non-melanoma/unspecified skin | 738 | 207 | 1.08 (0.98, 1.19) | 0.121 |  |  |
| All-cause mortality | Colorectal | 205 | 84 | 1.01 (0.88, 1.17) | 0.878 |  |  |
| All-cause mortality | Gynecologic | 416 | 57 | 0.91 (0.80, 1.04) | 0.180 |  |  |
| All-cause mortality | Melanoma | 191 | 45 | 1.04 (0.87, 1.23) | 0.680 |  |  |
| All-cause mortality | Hematologic | 123 | 44 | 0.80 (0.61, 1.06) | 0.116 |  |  |
| All-cause mortality | Lung | 73 | 36 | 0.95 (0.74, 1.22) | 0.687 |  |  |
| All-cause mortality | Urinary | 126 | 46 | 1.07 (0.87, 1.30) | 0.517 |  |  |
| All-cause mortality | Other/unknown | 368 | 111 | 0.94 (0.84, 1.05) | 0.263 |  |  |
| Cancer-specific mortality | Breast | 495 | 48 | 1.05 (0.94, 1.18) | 0.392 | <0.001 | 0.002 |
| Cancer-specific mortality | Prostate | 495 | 64 | 1.23 (1.01, 1.50) | 0.042 |  |  |
| Cancer-specific mortality | Non-melanoma/unspecified skin | 738 | 49 | 1.09 (0.93, 1.28) | 0.285 |  |  |
| Cancer-specific mortality | Colorectal | 205 | 23 | 0.84 (0.67, 1.07) | 0.159 |  |  |
| Cancer-specific mortality | Gynecologic | 416 | 12^*^ | 0.78 (0.56, 1.09) | 0.144 |  |  |
| Cancer-specific mortality | Melanoma | 191 | 11^*^ | 0.94 (0.73, 1.22) | 0.649 |  |  |
| Cancer-specific mortality | Hematologic | 123 | 30 | 0.86 (0.67, 1.11) | 0.257 |  |  |
| Cancer-specific mortality | Lung | 73 | 20 | 0.84 (0.61, 1.17) | 0.298 |  |  |
| Cancer-specific mortality | Urinary | 126 | 16^*^ | 1.20 (0.96, 1.51) | 0.113 |  |  |
| Cancer-specific mortality | Other/unknown | 368 | 42 | 0.76 (0.63, 0.92) | 0.005 |  |  |
| CVD mortality | Breast | 495 | 21 | 0.95 (0.78, 1.16) | 0.629 | 0.380 | 0.428 |
| CVD mortality | Prostate | 495 | 43 | 1.00 (0.82, 1.23) | 0.999 |  |  |
| CVD mortality | Non-melanoma/unspecified skin | 738 | 46 | 1.26 (1.01, 1.56) | 0.036 |  |  |
| CVD mortality | Colorectal | 205 | 21 | 1.19 (0.93, 1.53) | 0.167 |  |  |
| CVD mortality | Gynecologic | 416 | 15^*^ | 0.95 (0.77, 1.19) | 0.678 |  |  |
| CVD mortality | Melanoma | 191 | 13^*^ | 1.18 (0.85, 1.64) | 0.325 |  |  |
| CVD mortality | Hematologic | 123 | 5^*^ | 1.01 (0.75, 1.37) | 0.937 |  |  |
| CVD mortality | Lung | 73 | 3^*^ | 1.58 (0.85, 2.92) | 0.149 |  |  |
| CVD mortality | Urinary | 126 | 9^*^ | 0.89 (0.64, 1.24) | 0.494 |  |  |
| CVD mortality | Other/unknown | 368 | 21 | 1.04 (0.86, 1.26) | 0.703 |  |  |

*HRs were derived as linear contrasts from a pooled Cox model containing the continuous obesity indicator × cancer-group interaction, with common coefficients for the other covariates. They are not estimates from independently fitted fully adjusted models in each cancer group. Model 3 adjusted for Age (categorical), Sex, Race/ethnicity, Education level, PIR (categorical), Marital status, Alcohol status, Smoking status, Cancer group, and Years since first cancer diagnosis (continuous).*

*BRI estimates are reported per one-unit increase. All models incorporated WTMEC16YR, SDMVSTRA, and SDMVPSU. N and death counts are unweighted.*

** Fewer than 20 deaths: estimates require particular caution; this is a descriptive flag, not a universal validity threshold. All within-group P values are descriptive and unadjusted for multiple testing. Global interaction P values assess heterogeneity across all ten cancer groups; see Table S13 for multiplicity families.*

*Abbreviations: ABSI, a body shape index; BRI, body roundness index; BMI, body mass index; CI, confidence interval; CVD, cardiovascular disease; HR, hazard ratio; BH, Benjamini–Hochberg.*

**Table S12C.** Exploratory cancer-group-specific associations of BMI with mortality in survey-weighted Model 3.

| **Outcome** | **Cancer group** | **N** | **Events** | **HR (95% CI)** | **P value** | **P interaction** | **FDR q (9 tests)** |
| --- | --- | --- | --- | --- | --- | --- | --- |
| All-cause mortality | Breast | 495 | 133 | 0.98 (0.94, 1.01) | 0.153 | 0.763 | 0.763 |
| All-cause mortality | Prostate | 495 | 174 | 1.01 (0.95, 1.08) | 0.676 |  |  |
| All-cause mortality | Non-melanoma/unspecified skin | 738 | 207 | 1.01 (0.97, 1.05) | 0.719 |  |  |
| All-cause mortality | Colorectal | 205 | 84 | 1.01 (0.96, 1.07) | 0.630 |  |  |
| All-cause mortality | Gynecologic | 416 | 57 | 0.96 (0.91, 1.01) | 0.134 |  |  |
| All-cause mortality | Melanoma | 191 | 45 | 0.99 (0.92, 1.06) | 0.757 |  |  |
| All-cause mortality | Hematologic | 123 | 44 | 0.93 (0.83, 1.05) | 0.236 |  |  |
| All-cause mortality | Lung | 73 | 36 | 0.97 (0.89, 1.06) | 0.515 |  |  |
| All-cause mortality | Urinary | 126 | 46 | 1.02 (0.93, 1.11) | 0.734 |  |  |
| All-cause mortality | Other/unknown | 368 | 111 | 0.97 (0.94, 1.01) | 0.161 |  |  |
| Cancer-specific mortality | Breast | 495 | 48 | 1.01 (0.97, 1.05) | 0.693 | 0.012 | 0.053 |
| Cancer-specific mortality | Prostate | 495 | 64 | 1.07 (0.98, 1.17) | 0.115 |  |  |
| Cancer-specific mortality | Non-melanoma/unspecified skin | 738 | 49 | 1.02 (0.96, 1.08) | 0.578 |  |  |
| Cancer-specific mortality | Colorectal | 205 | 23 | 0.95 (0.87, 1.04) | 0.269 |  |  |
| Cancer-specific mortality | Gynecologic | 416 | 12^*^ | 0.87 (0.74, 1.03) | 0.107 |  |  |
| Cancer-specific mortality | Melanoma | 191 | 11^*^ | 0.98 (0.88, 1.09) | 0.720 |  |  |
| Cancer-specific mortality | Hematologic | 123 | 30 | 0.96 (0.85, 1.08) | 0.493 |  |  |
| Cancer-specific mortality | Lung | 73 | 20 | 0.95 (0.86, 1.05) | 0.323 |  |  |
| Cancer-specific mortality | Urinary | 126 | 16^*^ | 1.08 (0.99, 1.18) | 0.077 |  |  |
| Cancer-specific mortality | Other/unknown | 368 | 42 | 0.93 (0.86, 1.00) | 0.049 |  |  |
| CVD mortality | Breast | 495 | 21 | 0.93 (0.85, 1.02) | 0.126 | 0.198 | 0.296 |
| CVD mortality | Prostate | 495 | 43 | 0.98 (0.88, 1.08) | 0.618 |  |  |
| CVD mortality | Non-melanoma/unspecified skin | 738 | 46 | 1.06 (0.96, 1.17) | 0.221 |  |  |
| CVD mortality | Colorectal | 205 | 21 | 1.08 (0.97, 1.21) | 0.145 |  |  |
| CVD mortality | Gynecologic | 416 | 15^*^ | 0.96 (0.86, 1.07) | 0.508 |  |  |
| CVD mortality | Melanoma | 191 | 13^*^ | 1.03 (0.87, 1.22) | 0.746 |  |  |
| CVD mortality | Hematologic | 123 | 5^*^ | 0.91 (0.84, 0.99) | 0.022 |  |  |
| CVD mortality | Lung | 73 | 3^*^ | 1.26 (0.82, 1.94) | 0.298 |  |  |
| CVD mortality | Urinary | 126 | 9^*^ | 0.93 (0.82, 1.05) | 0.254 |  |  |
| CVD mortality | Other/unknown | 368 | 21 | 0.99 (0.93, 1.06) | 0.790 |  |  |

*HRs were derived as linear contrasts from a pooled Cox model containing the continuous obesity indicator × cancer-group interaction, with common coefficients for the other covariates. They are not estimates from independently fitted fully adjusted models in each cancer group. Model 3 adjusted for Age (categorical), Sex, Race/ethnicity, Education level, PIR (categorical), Marital status, Alcohol status, Smoking status, Cancer group, and Years since first cancer diagnosis (continuous).*

*BMI estimates are reported per 1 kg/m² increase. All models incorporated WTMEC16YR, SDMVSTRA, and SDMVPSU. N and death counts are unweighted.*

** Fewer than 20 deaths: estimates require particular caution; this is a descriptive flag, not a universal validity threshold. All within-group P values are descriptive and unadjusted for multiple testing. Global interaction P values assess heterogeneity across all ten cancer groups; see Table S13 for multiplicity families.*

*Abbreviations: ABSI, a body shape index; BRI, body roundness index; BMI, body mass index; CI, confidence interval; CVD, cardiovascular disease; HR, hazard ratio; BH, Benjamini–Hochberg.*

**Table S13.** Global cancer-group interaction tests for continuous obesity indices and mortality.

| **Outcome** | **Index** | **P interaction** | **FDR q (9 cancer-group tests)** | **FDR q (36 ABSI tests)** |
| --- | --- | --- | --- | --- |
| All-cause mortality | ABSI | 0.083 | 0.250 | 0.273 |
|  | BRI | 0.273 | 0.351 | — |
|  | BMI | 0.763 | 0.763 | — |
| Cancer-specific mortality | ABSI | 0.193 | 0.296 | 0.402 |
|  | BRI | <0.001 | 0.002 | — |
|  | BMI | 0.012 | 0.053 | — |
| CVD mortality | ABSI | 0.120 | 0.269 | 0.331 |
|  | BRI | 0.380 | 0.428 | — |
|  | BMI | 0.198 | 0.296 | — |

*Each P value is from a design-based global Wald test of the nine indicators × cancer-group interaction coefficients in Model 3. The three obesity indices were evaluated in separate models. Model 3 adjusted for Age (categorical), Sex, Race/ethnicity, Education level, PIR (categorical), Marital status, Alcohol status, Smoking status, Cancer group, and Years since first cancer diagnosis (continuous).*

*Two multiplicity families are distinguished: (1) all 36 ABSI subgroup interaction tests (12 subgroup factors × three outcomes; Table S9A); and (2) the nine exploratory cancer-group tests (three indices × three outcomes). A dash indicates that the test does not belong to the ABSI-only family. The nine-test correction is not study-wide multiplicity control.*

*For cancer-specific mortality, the exact BRI interaction P value was 0.000223542 and the nine-test BH q value was 0.002011880. The corresponding BMI values were P=0.011872709 and q=0.053427192. These analyses are post hoc and hypothesis-generating, especially for cancer groups with few events.*

*All models incorporated WTMEC16YR, SDMVSTRA, and SDMVPSU. N and death counts are unweighted.*

*Abbreviations: ABSI, a body shape index; BRI, body roundness index; BMI, body mass index; BH, Benjamini–Hochberg; CVD, cardiovascular disease.*

**Table S14.** Sensitivity analysis excluding participants reporting non-melanoma or unspecified skin cancer.

| **Outcome** | **N** | **Events** | **Index** | **HR (95% CI)** | **P value** |
| --- | --- | --- | --- | --- | --- |
| All-cause mortality | 2,492 | 730 | ABSI | 1.22 (1.10, 1.34) | <0.001 |
|  |  |  | BRI | 0.99 (0.94, 1.03) | 0.561 |
|  |  |  | BMI | 0.98 (0.96, 1.00) | 0.054 |
| Cancer-specific mortality | 2,492 | 266 | ABSI | 1.09 (0.92, 1.30) | 0.305 |
|  |  |  | BRI | 0.97 (0.88, 1.06) | 0.507 |
|  |  |  | BMI | 0.99 (0.95, 1.02) | 0.463 |
| CVD mortality | 2,492 | 151 | ABSI | 1.50 (1.21, 1.87) | <0.001 |
|  |  |  | BRI | 1.03 (0.95, 1.13) | 0.451 |
|  |  |  | BMI | 0.99 (0.96, 1.03) | 0.682 |

*A total of 738 participants in the non-melanoma/unspecified skin cancer group were excluded, leaving 2,492 participants, including 730 all-cause, 266 cancer-specific, and 151 CVD deaths. Participants reporting melanoma were retained.*

*These sensitivity models used the main Model 3 specification without the indicator × cancer-group interaction. Cancer-group adjustment was retained among the remaining participants. Model 3 adjusted for Age (categorical), Sex, Race/ethnicity, Education level, PIR (categorical), Marital status, Alcohol status, Smoking status, Cancer group, and Years since first cancer diagnosis (continuous).*

*Estimates are reported per 1-SD increase in ABSI, per one-unit increase in BRI, and per 1 kg/m² increase in BMI. All models incorporated WTMEC16YR, SDMVSTRA, and SDMVPSU. N and death counts are unweighted. P values are nominal and not adjusted for multiple testing.*

*Abbreviations: ABSI, a body shape index; BRI, body roundness index; BMI, body mass index; CI, confidence interval; CVD, cardiovascular disease; HR, hazard ratio.*

**Figure S1.** Flowchart of Participant Selection.

**Figure S2.** Survey-weighted Kaplan-Meier survival curves by ABSI quartile, BRI quartile, and BMI category. Survey-weighted log-rank P values were: ABSI, P<0.0001 for all three outcomes; BRI, P=0.002 for all-cause, P=0.673 for cancer, and P=0.001 for CVD mortality; BMI, P=0.092, P=0.607, and P=0.885, respectively. Abbreviations: ABSI, a body shape index; BRI, body roundness index; BMI, body mass index; CVD, cardiovascular disease.

**Figure S3.** Directed acyclic graph illustrating the assumed relationships underlying covariate selection. Model 3 adjusts for sociodemographic, behavioral, and cancer-history variables selected on epidemiologic grounds as potential confounders. Hypertension, diabetes, and CVD history are treated as potential cardiometabolic intermediates or mixed-role variables and are included only in the Model 4 sensitivity analyses.

**Figure S4.** Complete-case and covariate-only multiple-imputation estimates for continuous obesity indices in Models 3 and 4. Squares indicate complete-case estimates (N=3,230), circles indicate multiple-imputation estimates (N=3,714), and horizontal lines indicate 95% confidence intervals. ABSI is scaled per 1 SD; BRI per 1 unit; BMI per 1 kg/m².

**Figure S5.** Exploratory cancer-group-specific associations of ABSI with mortality. HRs and 95% CIs were estimated from survey-weighted Model 3 with an ABSI × cancer-group interaction and common coefficients for the other covariates. ABSI uses the same pooled z-score scale as the main analysis. Global interactions were assessed by design-based Wald tests; BH q values refer to all 36 ABSI subgroup tests. Within-group P values are unadjusted. * Fewer than 20 deaths; interpret cautiously. Arrows indicate CIs extending beyond the plotted axis. Abbreviations: ABSI, a body shape index; BH, Benjamini–Hochberg; CI, confidence interval; CVD, cardiovascular disease; HR, hazard ratio.
